# RNA virus discoveries in the electric ant, *Wasmannia auropunctata*

**DOI:** 10.1007/s11262-023-01969-1

**Published:** 2023-02-02

**Authors:** Steven M. Valles, Chaoyang Zhao, Adam R. Rivers, Ryo L. Iwata, David H. Oi, Dong H. Cha, R. Max Collignon, Nastassja A. Cox, Gary J. Morton, Luis A. Calcaterra

**Affiliations:** 1 Center for Medical, Agricultural and Veterinary Entomology, USDA-ARS, 1600 SW 23rd Drive, Gainesville, FL USA; 2 Genomics and Bioinformatics Research Unit, USDA-ARS, 1600 SW 23rd Drive, Gainesville, FL USA; 3 Daniel K. Inouye U.S. Pacific Basin Agricultural Research Center, USDA-ARS, 64 Nowelo St, Hilo, HI USA; 4 National Electric Ant Eradication Program, Department of Agriculture and Fisheries, Biosecurity Queensland, 21–23 Redden Street, Cairns, QLD 4870 Australia; 5 Fundación para el Estudio de Especies Invasivas, Bolívar 1559, B1686EFA, Hurlingham, Buenos Aires, Argentina

**Keywords:** Electric ant, Little fire ant, *Wasmannia auropunctata*, Virome, RNA virus, Metagenomics

## Abstract

Despite being one of the most destructive invasive species of ants, only two natural enemies are known currently for *Wasmannia auropunctata,* commonly known as the electric ant or little fire ant. Because viruses can be effective biological control agents against many insect pests, including ants, a metagenomics/next-generation sequencing approach was used to facilitate discovery of virus sequences from the transcriptomes of *W. auropunctata*. Five new and complete positive sense, single-stranded RNA virus genomes, and one new negative sense, single-stranded RNA virus genome were identified, sequenced, and characterized from *W. auropunctata* collected in Argentina by this approach, including a dicistrovirus (Electric ant dicistrovirus), two polycipiviruses (Electric ant polycipivirus 1; Electric ant polycipivirus 2), a solinvivirus (Electric ant solinvivirus), a divergent genome with similarity to an unclassified group in the *Picornavirales* (Electric ant virus 1), and a rhabdovirus (Electric ant rhabdovirus). An additional virus genome was detected that is likely Solenopsis invicta virus 10 (MH727527). The virus genome sequences were absent from the transcriptomes of *W. auropunctata* collected in the USA (Hawaii and Florida). Additional limited field surveys corroborated the absence of these viruses in regions where the electric ant is invasive (the USA and Australia). The replicative genome strand of four of the viruses (Electric ant polycipivirus 2, Electric ant solinvivirus, Electric ant virus 1, and Solenopsis invicta virus 10 (in the electric ant) was detected in Argentinean-collected *W. auropunctata* indicating that the ant is a host for these viruses. These are the first virus discoveries to be made from *W. auropunctata*.

**Supplementary Information:**

The online version contains supplementary material available at 10.1007/s11262-023-01969-1.

## Introduction

*Wasmannia auropunctata* (also known as the electric ant or little fire ant) is an invasive myrmicine tramp ant species native to Central and South America that is now well established throughout tropical and sub-tropical regions of the world, including the USA (specifically Texas, Florida, and Hawaii) [10, 30, 45, 46]. Considered one of the world’s 100 most invasive species [26], the electric ant is a significant agricultural pest because it stings farm workers and enhances certain hemipteran populations, which saps plants of nutrients and vigor, and increases the occurrence of viral and fungal infections [45]. The electric ant also exhibits direct and indirect negative ecological impacts on local flora and fauna [2, 45].

*W. auropunctata* exhibits a rare and facultative reproductive polymorphism whereby colonies may be formed sexually or clonally [9]. Sexual reproduction (i.e., haplodiploidy [15]) is characterized by fertilized eggs producing female queens and workers, and unfertilized haploid eggs producing arrhenotokous males. Clonal reproduction is characterized by female queens produced by thelytokous parthenogenesis; haploid males are genetically identical to their father, and female workers are produced sexually [9]. Interestingly, the shift from sexual to clonal reproduction has been shown to have occurred within the native range and not introduced regions as is typically the case [10, 11]. Sexual reproduction in electric ant is rare in introduced areas [8] and intra-specific aggression among introduced populations is not observed [7]. In addition, clonally reproducing electric ants better tolerate higher temperatures associated with human-modified habitats [11]. These characteristics suggest that clonally reproducing electric ants are better adapted to colonize new areas and likely contribute to their invasive success [11].

The destructive impact of *W. auropunctata* observed in introduced areas does not appear to occur in its native range [40], which may be attributed to a loss of natural enemies during founding possibly coincident with the reproductive adaptation. While support for this hypothesis is scant, it is not completely absent. For example, clonal populations of *W. auropunctata* have lost the *Wolbachia* bacterial infection characteristic of sexually reproducing populations [35]. Wetterer and Porter [45] recommended quarantine efforts, detection, and classical biological control efforts to discover, evaluate, and release natural enemies in introduced populations of *W. auropunctata* to provide sustainable control of the ant. Unfortunately, despite its serious pest status, the only natural enemies known for *W. auropunctata* include the eucharitid wasp, *Orasema minutissima* [28], and an army ant predator, *Neivamyrmex compressinodis* [23].

The dearth of natural enemies known from *W. auropunctata* prompted our investigation to examine the virome of this ant. Because viruses can be effective biological control agents against many insect pests [22], including ants [43], our primary objective was to employ a metagenomics/next-generation sequencing approach [12] to facilitate discovery of virus sequences (viruses by proxy) from the transcriptomes of *W. auropunctata* collected from areas within the native and introduced ranges. Viral sequences identified by this method were re-sequenced in entirety and the field prevalence of each viral sequence in *W. auropunctata* was compared between native and introduced regions. Finally, host status of identified virus genome sequences was evaluated by detection of the replicative genome strand in *W. auropunctata*.

## Materials and methods

### *Wasmannia auropunctata* collections for library preparation

Samples of adult worker *W. auropunctata* ants (~ 100–500/sample) or queens (~ 5/sample) were collected from field locations in Argentina (2019–2021), where the ant is native, and the USA (2020), where the ant is invasive, by aspiration, forceps, or at food lures and placed immediately in 2–5 ml of DNA/RNA shield (Zymo Research, Irvine, California) until they could be processed for RNA extraction.

In Argentina, ant samples were collected from 39 locations from across the country spanning an area of approximately 7 × 10^7^ hectares. Samples were grouped roughly by geographic location/collection date and labeled ARG1, ARG2, ARG3, and ARG4 (Supplementary Table 1). Group ARG1 included 10 samples of worker ants collected from colonies along the Paraná River from Buenos Aires to Posadas, Misiones province. Group ARG2 included 9 samples of worker ants collected from 1 × 10^6^ hectares around the Posadas region. Groups ARG3 and ARG4 included 9 and 11 samples of worker ants, respectively, collected from within Buenos Aires.

In the USA, ant samples were collected from locations in Florida and Hawaii. Florida samples were collected in Gainesville, Alachua County, and labeled groups FL1 and FL2 (Supplementary Table 1). Group FL1 included 6 samples of worker ants and Group FL2 included 4 samples of queens collected on the University of Florida campus.

Hawaii samples were collected from 3 locations on the island of Hawaii and labeled groups HI1, HI2, and HI3 (Supplementary Table 1). Group HI1 included 5 samples of worker ants from sites in Papaikou, on the eastern side of the island. Group HI2 included 5 samples of workers collected from Hilo, on the eastern side of the island. Group HI3 included 5 samples of workers collected on the western side of the island in Captain Cook.

### *Wasmannia auropunctata* collections for field evaluation

After sequencing, assembly, and virus genomes were established, additional field collections of *W. auropunctata* were made to evaluate their field presence and prevalence using RT-PCR from pooled collections of ants (*n* = 10 to 50/sample). Collections were made from the USA (Florida, Hawaii), Australia, and Argentina. For each sample evaluated, a positive control (RNA from a gene library) and non-template negative control were included. In addition, to verify the integrity of the RNA, each sample was also reverse transcribed, and PCR amplified for an internal, ant-specific gene (i.e., Actin). Oligonucleotide primers for all reactions are listed in Supplementary Table 2.

### RNA preparation

Total RNA was extracted from worker ants and queens using the Trizol method followed by the PureLink RNA Mini Purification Kit according to the manufacturer’s instructions (Thermo Fisher Scientific, Waltham, MA). RNA quality of each preparation was assessed by microfluidic analysis on an Agilent 2100 Bioanalyzer (Agilent, Cary, NC) using the RNA 6000 Nano kit according to the manufacturer’s instructions. Total RNA was submitted to Novogene Corporation Inc. (University of California, CA) for mRNA purification with oligo dT enrichment, rRNA removal with Illumina Ribo-Zero rRNA depletion kit (Illumina, San Diego, CA), library preparation, and Illumina RNA sequencing using the MiSeq chemistry (MiSeq Reagent kit, Illumina, San Diego, CA). cDNA was synthesized using mRNA template and random hexamers primers, after which a custom second-strand synthesis buffer (Illumina), dNTPs, RNase H, and DNA polymerase I were added to initiate the second-strand synthesis. Sequence adapters were ligated to the cDNA, the double-stranded cDNA library was completed by size selection and PCR enrichment. The qualified libraries were sequenced on an Illumina (MiSeq chemistry) sequencer after pooling according to its effective concentration and expected data volume. Paired reads were obtained.

### Library preparation and sequencing

Nine libraries were created from pooled samples corresponding to their collection site (ARG1, ARG2, ARG3, ARG4 (Argentina); FL1, FL2 (USA, Florida); HI1, HI2, and HI3 (USA, Hawaii)). Metadata for each library are summarized in Supplementary Table 1. The caste and approximate number of ants used for RNA preparation of each library were as follows: ARG1 (*n* ≈ 1000 worker ants); ARG2 (*n* ≈ 900 worker ants); ARG3 (*n* ≈ 900 worker ants); ARG4 (*n* ≈ 1100 worker ants); FL1 (*n* ≈ 500 worker ants); FL2 (*n* ≈ 20 queens); HI1 (*n* ≈ 600 workers); HI2 (*n* ≈ 600 workers); and HI3 (*n* ≈ 600 workers).

Quality control for total RNA purified from each of the pooled groups was analyzed by agarose gel electrophoresis to estimate RNA degradation and possible contamination followed by evaluation of RNA for integrity and quantification on an Agilent 2100 bioanalyzer. mRNA was enriched using oligo(dT) beads and ribosomal RNA was removed using Ribo-Zero (Illumina, Inc., San Diego, CA). The mRNA was fragmented randomly, and cDNA was synthesized using the mRNA template and random hexamer oligonucleotide primers. Second-strand synthesis was completed with second-strand synthesis buffer (Illumina), dNTPs, RNase H, and DNA polymerase I. After a series of terminal repair steps, an adaptor was ligated to the double-stranded cDNA, which was subsequently size selected and enriched by PCR. The qualified libraries were sequenced on Illumina processors. Sequence data (raw reads and error) for each library are summarized in Supplementary Table 3.

### Metagenomic analysis

The paired-end sequence data were processed with BBtools BBduk version 38.79b (https://sourceforge.net/projects/bbmap/) to remove the sequences of known laboratory contaminants and trim sequencing adapters from the remaining sequences. Reads were combined and sorted by shared Kmer content using BBtools Clumpify to speed up assembly and improve compression. Spades version 3.14.0 was run in meta mode to create metagenomics contigs. Contigs longer than 2000 nt were annotated with Diamond Version 2.0.6 against the nr database and then functional and taxonomic assignment were accomplished using Megan version 6.18.4. Viral contigs were used as templates to create complete viral genomes by Sanger sequencing (see virus genome re-sequencing below). To estimate the abundance of the viruses in the samples, the initial viral contigs were replaced with the complete genomes and all reads were mapped from each sample back to the virus genomes and metagenomic contigs using BBtools bbmap version 38.79 (https://sourceforge.net/projects/bbmap/). Across all gene libraries, the mean sequence length obtained was 241 ± 1.3 nucleotides, and the mean proportion of sequences mapping to Formicidae was 66.2 ± 5.9%. These statistics are provided for each library in Supplementary Table 3.

### Data availability

The metagenomics sequence data for this project are available at the National Center for Biotechnology Information under Bioproject accession PRJNA658153, which includes 9 associated Biosample accessions with MIMS metagenome/environmental host-associated metadata and 9 SRA accessions with sequence data. The individual-annotated Sanger-derived viral genomes are deposited under NCBI accessions: Electric ant dicistrovirus (OP518023), Electric ant polycipivirus 1 (OP518021), Electric ant polycipivirus 2 (OP518022), Electric ant solinvivirus (OP518024), Electric ant virus 1 (OP518025), Solenopsis invicta virus 10 in electric ant (OP518026), and Electric ant rhabdovirus (OP518027).

### Detection of replicative genome

For each of the seven virus genomes identified, additional tests were conducted to detect the replicative genome strand in *W. auropunctata*. Active virus replication was evaluated by detection of the replicative genome strand of each of the viruses by the modified tagged method of Craggs et al. [5]. Total RNA (50 ng) was mixed with 10-mM dNTPs and 1-µM tagged reverse oligonucleotide primer (see Supplementary Table 2) and heated to 65 °C for 5 min. First-strand buffer and Superscript reverse transcriptase were added, and the reaction mixture was incubated at 55 °C for 1 h before inactivating the RT at 70 °C for 15 min. Unincorporated cDNA oligonucleotides were digested with 10 units of Exonuclease I (New England Biolabs, Ipswich, MA) at 37 °C for 1 h. The reaction was terminated by heating to 80 °C for 20 min. PCR was subsequently conducted with replicative-strand-specific cDNA as template. The reaction was conducted in a 25-μl volume containing 2-mM MgCl_2_, 200-μM dNTP mix, 0.5 units of Platinum Taq DNA polymerase, 0.2 μM of each oligonucleotide primer (gene specific and TAG 5'GGCCGTCATGGTGGCGAATAA), and 5 μl of the cDNA preparation. The temperature cycling program was 1 cycle at 94º C for 2 min, 35 cycles of 94 ºC for 15 s, 59 ºC for 15 s, 68 ºC for 30 s, and 1 cycle of 68 ºC for 5 min. PCR products were separated on an agarose gel (1%) and visualized by SYBR-safe staining.

### Virus genome re-sequencing

Seven partial RNA virus genomes were assembled from the Illumina-derived MiSeq sequences. These sequences were used as templates to design oligonucleotide primers to provide complete overlapping coverage of each genome. The genomes of each virus were RT-PCR amplified in ~ 1200 nucleotide sections from RNA obtained from the corresponding gene library. Amplicons were cloned into pCR4 vector and sequenced by the Sanger method. The termini of each genome were obtained with 5' and 3' rapid amplification of cDNA ends (RACE). For 3' RACE, cDNA was synthesized with the GeneRacer Oligo dT primer (Invitrogen, Carlsbad, CA) and PCR subsequently conducted with the GeneRacer 3' primer and a gene-specific primer (Supplementary Table 2). For 5' RACE, cDNA was synthesized with a gene-specific oligonucleotide primer and PCR was later completed with a nested gene-specific primer and the GeneRacer Abridged Anchor Primer (Supplementary Table 2). Amplicons generated during RACE were also cloned into pCR4 vector and submitted for Sanger sequencing. Genomes were assembled with the CAP3 program and a minimum of threefold genome coverage was obtained.

### Phylogenetic analysis

To gain gross phylogenetic relationships and possible taxonomic placement of the *W. auropunctata* virus genome sequences (except the rhabdovirus) within the picorna-like virus superfamily, preliminary phylogenetic analysis was first conducted using the conserved amino acid RNA-dependent RNA polymerase (RdRp) sequences of 75 phylogenetically widespread viruses identified by Koonin et al. 2008 [19]. For this initial analysis, RdRp amino acid regions were aligned with MUSCLE [6] and subsequently analyzed by the Maximum Likelihood method with the JTT matrix-based model [17] to infer an evolutionary relationship [20] (see Supplementary Fig. 1).

Based on the preliminary phylogeny of the six picorna-like viruses (Supplementary Fig. 1), three separate, more specific, phylogenetic analyses were conducted to suggest taxonomic placement of each virus genome. In addition to inclusion of some virus groups identified in the preliminary phylogeny, Blastp analysis [1] against the National Center for Biotechnology Information (NCBI) database was used to identify related sequences in the refined phylogenetic analyses. The top related sequences were chosen for inclusion in the analysis. Four separate phylogenetic analyses were conducted to place the seven virus genomes identified. The translated “L” open reading frame (ORFL; transcription and replication proteins) was used to analyze the Electric ant rhabdovirus (EARV); translated ORF5 (non-structural proteins) was used to analyze the policipiviruses, Electric ant polycipivirus 1 (EAPV1), and Electric ant polycipivirus 2 (EAPV2); and translated ORF1 (non-structural proteins) was used to analyze the Electric ant dicistrovirus (EADV). The RdRp protein sequence was used to conduct the phylogenetic analysis for the Electric ant virus 1 (EAV1), Solenopsis invicta virus 10 in electric ant (SINV10 in EA), and Electric ant solinvivirus (EASV). EAV1 and SINV10 in EA grouped within clades of virus sequences that have not been classified currently and we wanted to illustrate clustered relationships as broadly as possible. In this case, we included representative RdRp sequences from all families within the *Picornavirales*.

All sequences were first aligned using MUSCLE included in MEGA (version 11) with default settings [21]. Poorly aligned or highly divergent regions were removed using TrimAL (version 1.3) available on the Phylemon 2 server ( http://phylemon2.bioinfo.cipf.es/) with ‘Automated 1’ as the selected method. The trimmed sequence alignments were then uploaded to MEGA to determine, according to the Bayesian Information Criterion (BIC), the optimal substitution models, which were ‘LG + G + I + F,’ ‘LG + G + I,’ ‘LG + G + I,’ and ‘LG + G’ for the *Rhabdoviridae* (ORFL), *Polycipiviridae* (ORFL5), *Dicistroviridae* (ORF1), and *Picornavirales* (RdRp) runs, respectively. Phylogenetic trees were constructed by testing 500 replicates using the maximum likelihood method on MEGA and visualized using FigTree (version 1.4.4).

### Maps

Maps used to illustrate collection sites in Argentina were generated using the Google maps application (Google, no date); retrieved September 8, 2022, from https://www.google.com/maps/@-30.0810879,-60.982223,7z/data=!3m1!4b1!4m2!6m1!1s1HDGbji7QInGfDNJo27pZEOv3BtnYfJk?hl=en).

## Results

Seven complete RNA virus genomes were discovered from the metatranscriptome of *W. auropunctata* field colonies collected from across Argentina (Table 1). Six of these sequences were unique and not detected in GenBank database searches. However, one sequence was detected in GenBank exhibiting 99% polyprotein identity with SINV10 (MH727527). For clarity, this virus is henceforth referred to as SINV10 in electric ant [SINV10 in EA]). Conspicuously, none of the virus sequences were observed in gene libraries created from *W. auropunctata* collected in the USA (i.e., Florida and Hawaii). Six of the virus genomes exhibited sequence identity, domain motifs, and genome architecture characteristics consistent with positive sense, single-stranded RNA viruses of the *Picornavirales* [24], including a dicistrovirus (Electric ant dicistrovirus, EADV), two polycipiviruses (Electric ant polycipivirus 1, EAPV1; Electric ant polycipivirus 2, EAPV2), a solinvivirus (Electric ant solinvivirus, EASV), and two divergent genomes with similarity to unclassified groups in the *Picornavirales* (Electric ant virus 1, EAV1; Solenopsis invicta virus 10 in electric ant, SINV10 in EA). The genome sequences possessed NTPase domains containing the conserved Walker A motif (Gx_4_GK[S/T]) indicative of helicase function and an RdRp motif characteristic of viruses in this order [29]. They also encoded single to multiple large ORFs in the sense orientation only. One genome sequence (Electric ant rhabdovirus, EARV) exhibited sequence identity and genome characteristics consistent with negative sense, single-stranded RNA viruses in the *Rhabdoviridae*. Proposed virus and species names, the tentative taxonomic placement, genome type, and GenBank accession numbers are summarized in Table 1.

**Table 1:** Virus name (abbreviation), species, and characteristics of virus genome sequences from *Wasmannia auropunctata*

Virus	Species	Family	Genome	Genome length	GenBank accession
Electric ant polycipivirus 1 (EAPV1)	*Sopolycivirus calcatterai*	*Polycipiviridae*	PS, SS, RNA	11,287	OP518021
Electric ant polycipivirus 2 (EAPV2)	*Sopolycivirus riversi*	*Polycipiviridae*	PS, SS, RNA	11,252	OP518022
Electric ant dicistrovirus (EADV)	*Triatovirus electrico*	*Dicistroviridae*	PS, SS, RNA	10,197	OP518023
Electric ant solinvivirus (EASV)	*Invictavirus electrico*	*Solinviviridae*	PS, SS, RNA	11,346	OP518024
Electric ant virus 1 (EAV1)	*Electric ant virus 1*	*Incertae sedis*	PS, SS, RNA	10,233	OP518025
Solenopsis invicta virus 10 [in electric ant] (SINV10 in EA)	*Solenopsis invicta virus 10*	*Incertae sedis*	PS, SS, RNA	10,979	OP518026
Electric ant rhabdovirus (EARV)	*Alphahymrhavirus electrico*	*Rhabdoviridae*	NS, SS, RNA	12,034	OP518027

**Fig. 1:**
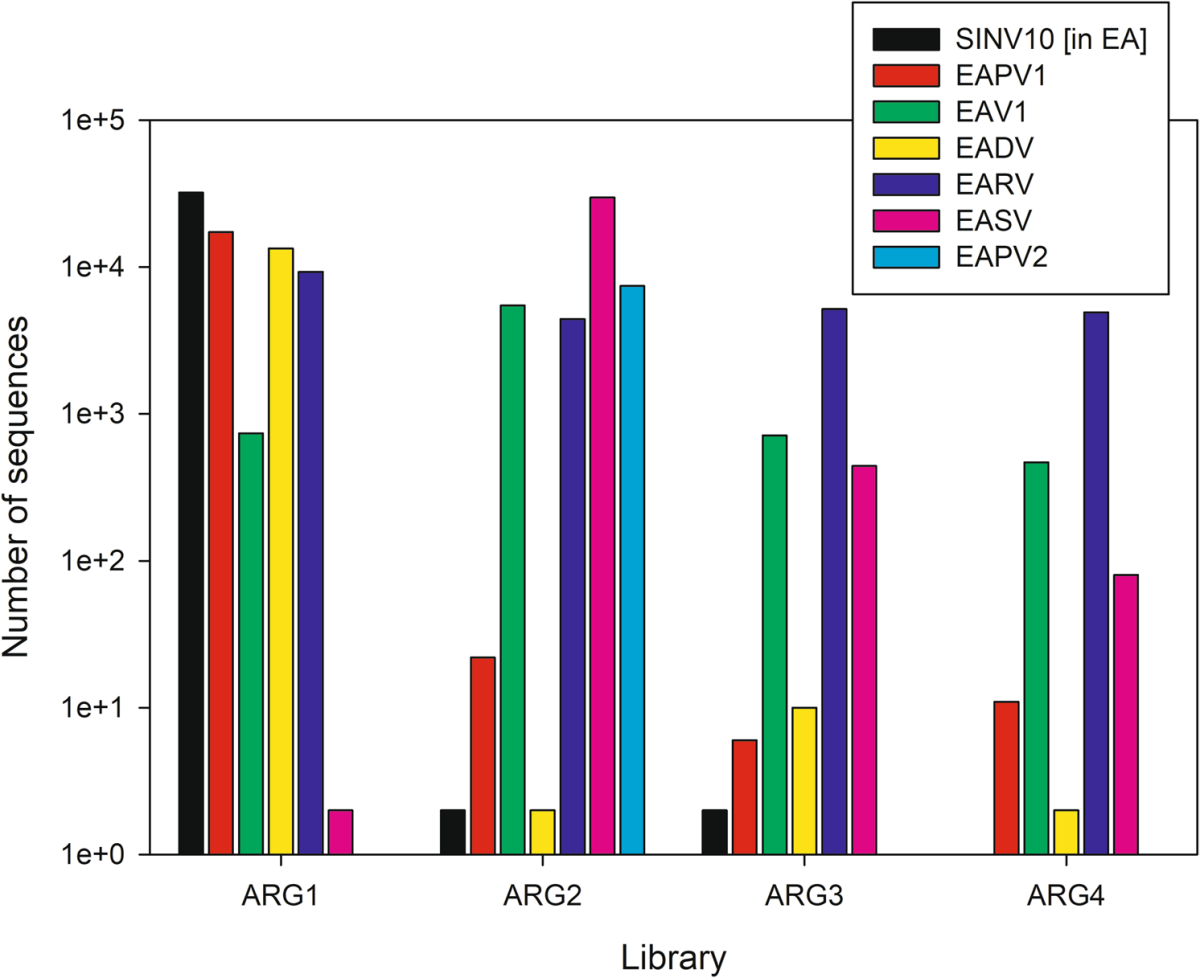
Number of unambiguous virus sequence reads for each virus genome sequence detected within each gene library. No matches were observed for USA-derived *Wasmannia auropunctata* gene libraries: HI1, HI2, HI3, FL1, and FL2. The total number of virus sequences after non-virus sequences were removed for each library was ARG1 (72,648), ARG2 (46,967), ARG3 (6326), and ARG4 (5452)

Virus sequences varied by gene library with ARG2 exhibiting the greatest virus diversity, which included representatives of all the virus sequences (i.e., EAV1, SINV10 in EA, EAPV1, EAPV2, EADV, EASV, and EARV) (Fig. 1). EAPV2 was not detected in library ARG1, ARG3, or ARG4. SINV10 in EA was not detected in library ARG4. The number of sequences detected also varied by library with SINV10 in EA most prevalent in ARG1, EASV most prevalent in ARG2, and EARV most prevalent in ARG3 and ARG4. Again, none of the virus sequences were detected in the USA metatranscriptome sources of *W. auropunctata*.

### Electric ant dicistrovirus (EADV)

**Fig. 2:**
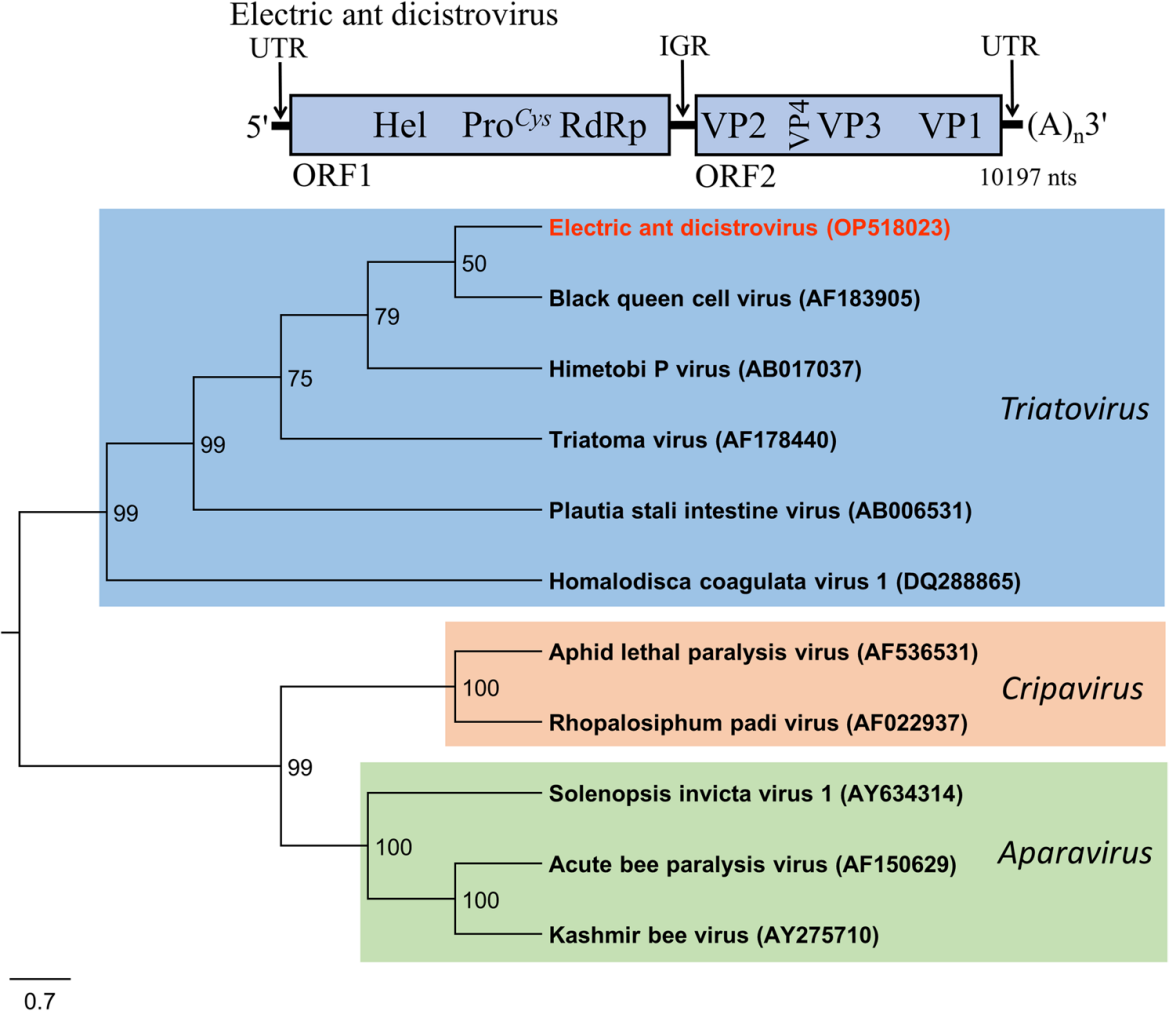
Diagrammatic representation of the predicted genome map of Electric ant dicistrovirus (top) and unrooted phylogenetic tree (bottom) for Electric ant dicistrovirus generated from the amino acid sequence of the predicted polyprotein of ORF1 (non-structural proteins). Numbers on the internal nodes represent the bootstrap values (500 replicates). The three established dicistrovirus genera, *Triatovirus*, *Cripavirus*, and *Aparavirus*, are highlighted by a unique shading color. Legend for the number of substitutions per site is shown at the bottom left and GenBank accession numbers follow each virus name. ORFs are illustrated as blocks relative to the genome (line), and their position relative to the genome represents the reading frame (below line = reading frame [rf] 1; mid-line = rf 2; above line = rf 3)

The RNA genome of EADV (proposed species *Triatovirus electrico*) was 10,197 nucleotides (nts) in length, excluding the polyadenylated 3' terminus. The genome included two large non-overlapping ORFs in the sense orientation consistent with dicistroviruses, ORF1 (1818 aa) and ORF2 (1226 aa canonical start; 1279 predicted non-canonical start) (Fig. 2). Blastx [1] analysis revealed significant identity to non-structural (ORF1) and structural (ORF2) proteins of viruses in the *Dicistroviridae*. The non-structural proteins exhibited significant identity to viruses within the *Triatovirus* genus, including Black queen cell virus (BQCV) of honey bees (62% genome coverage: 38% sequence identity) [41]. ORF1 encoded a polyprotein with significant identity to helicase (aa 2224–2580), 3C-like cysteine protease (aa 1090–1256, active site: ***C***G at aa 1234), and RdRp proteins involved in genome replication. Phylogenetic analysis of the non-structural polyprotein corresponded with dicistrovirus characteristics by placement of EADV within the *Dicistroviridae* family and *Triatovirus* genus with strong bootstrap (99%) support for the cluster of *Triatovirus* members, including EADV [41] (Fig. 2).

EADV contains the highly conserved domain 2 regions of the intergenic internal ribosomal entry site (IGR-IRES) (i.e., AUUU [genome position 6052] and the loop sequence CAGCC [genome position 6104]), which are important for efficient translation and ribosome-binding affinity in dicistroviruses [16, 31, 32]. The IGR-IRES of dicistroviruses directs translation of ORF2 (capsid proteins) at a non-AUG start site [31]. The EADV ORF2 initiation site was inferred from sequence alignment of other triatoviruses and found most likely to occur at nucleotide position 6180 encoding an alanine at the first position (ASINNQ…) [18, 31].

Triatoviruses also possess a conserved ternary motif (DDF) at the carboxyl end of VP1 and VP3, which are involved in autoproteolysis [39]. EADV possesses the DDF triad at amino acid 570 of ORF 2 [within VP3]), but the second DDF triad in VP1 of Triatoma virus is not present in EADV. However, the sequence DDM (aa 1132) is present at the carboxyl end of VP1 of EADV and aligns with the DDF sequence of Triatoma virus, also at the carboxyl end of VP1. BQCV, also a triatovirus, similarly possesses this DDM sequence at the carboxyl end of VP1.

### Electric ant polycipivirus 1 and 2 (EAPV1 and EAPV2)

**Fig. 3:**
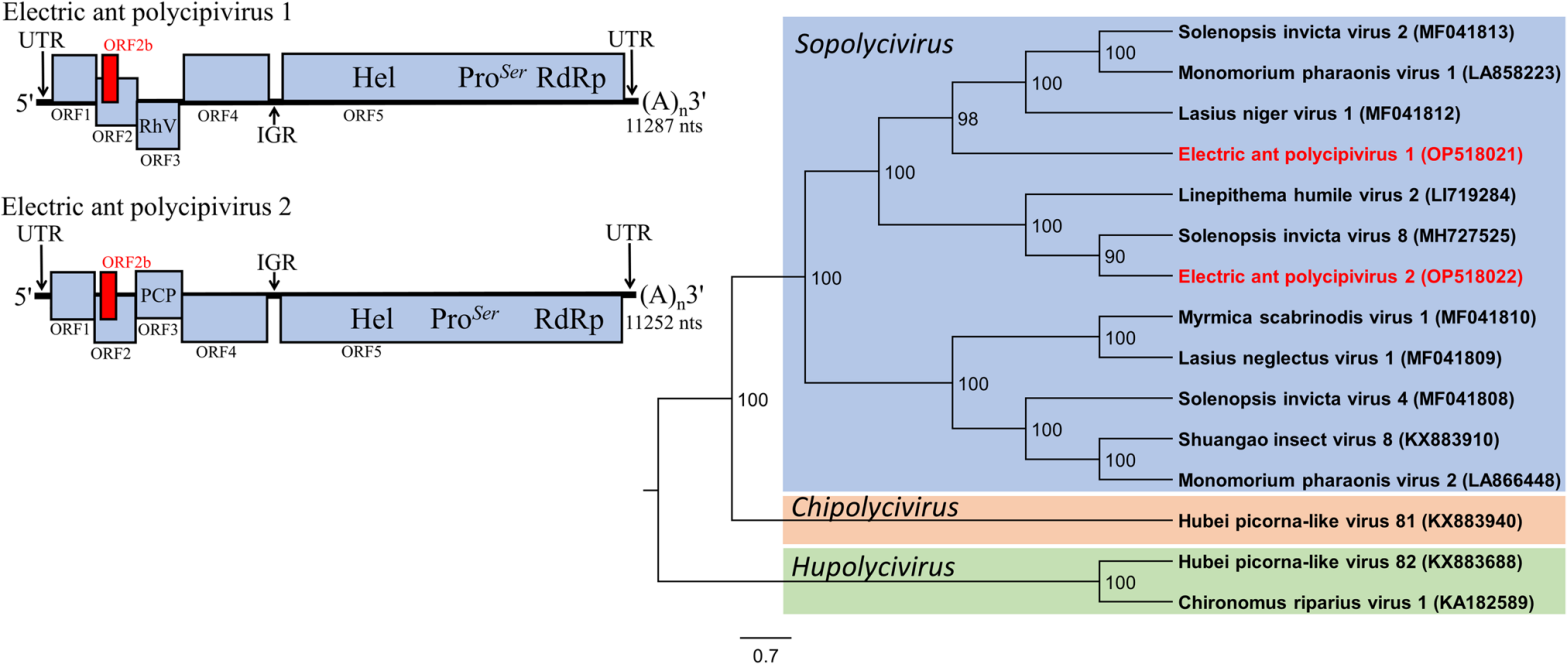
Diagrammatic representation of the predicted genome map of Electric ant polycipivirus 1 and Electric ant polycipivirus 2 (left). Unrooted phylogenetic tree for Electric ant polycipivirus 1 and Electric ant polycipivirus 2 was generated using the amino acid sequence from ORF5 (non-structural proteins). Numbers on the internal nodes represent the bootstrap values (500 replicates). The three established polycipivirus genera, *Sopolycivirus*, *Chipolycivirus*, and *Hupolycivirus* are highlighted by a unique shading color. Legend for the number of substitutions per site is shown at the bottom left and GenBank accession numbers follow each virus name. ORFs are illustrated as blocks relative to the genome (line), and their position relative to the genome represents the reading frame (below line = reading frame [rf] 1; mid-line = rf 2; above line = rf 3)

The EAPV1 (proposed species *Sopolycivirus calcatterai*) and EAPV2 (proposed species *Sopolycivirus riversi*) genome sequences were 11,287 and 11,252 nts, respectively, each with 5 non-overlapping ORFs in the sense direction and one overlapping ORF2b (over ORF2) predicted (Fig. 3). ORFs 1, 3, and 4 exhibited significant sequence identity with RNA virus capsid proteins; ORF 5 exhibited significant sequence identity with helicase (aa 725–833; 729–837), protease (aa 1433–1589; 1500–1654), and RdRp (aa 1827–2094; 1900–2172) of RNA viruses from the *Polycipiviridae*. A portion of the EAPV1 ORF3 also exhibited identity with the rhinovirus-like (RhV) capsid protein (aa 106–237). The protease of both EAPV1 and EAPV2 appear to be serine proteases as the active site of each possesses a serine (ORF5 aa 1571 and 1633, for EAPV1 and EAPV2, respectively). Phylogenetic analysis of the ORF5 polyprotein clusters EAPV1 and EAPV2 within the new family *Polycipiviridae* and genus *Sopolycivirus* [33] (Fig. 3). The unique polycistronic genomic architecture supports this taxonomic placement. ORF5 of EAPV1 exhibited 98% genome coverage and 46% sequence identity with Lasius niger virus 1 (MF041812), while EAPV2 exhibited 96% genome coverage and 37% sequence identity with SINV8 (MH727525).

### Electric ant solinvivirus (EASV)

**Fig. 4:**
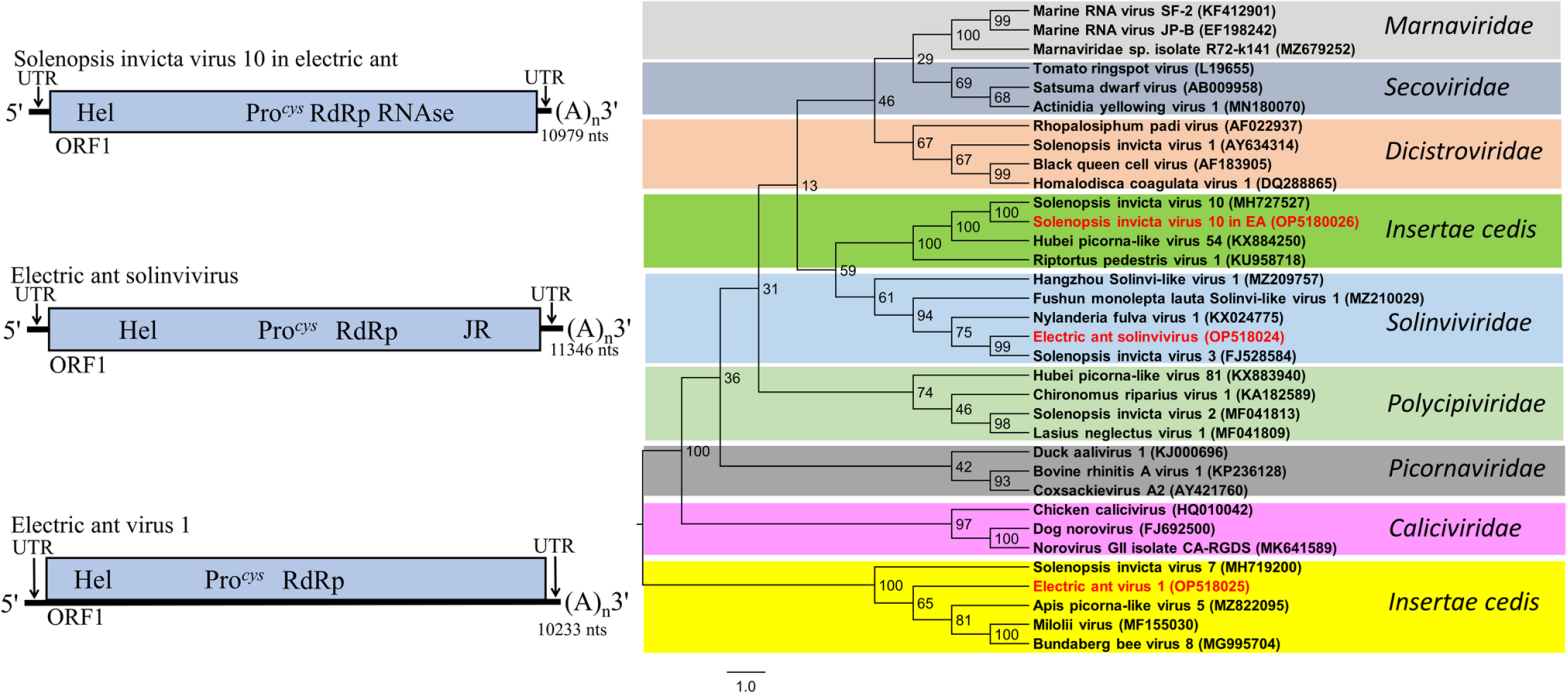
Diagrammatic representation of the predicted genome map of Electric ant solinvivirus, Electric ant virus 1, and Solenopsis invicta virus 10 in electric ant [EA] (left). Phylogenetic tree (unrooted) for Electric ant solinvivirus, Electric ant virus 1, and Solenopsis invicta virus 10 in EA were generated using the RNA-dependent RNA polymerase amino acid sequences. Numbers on the internal nodes represent the bootstrap values (500 replicates). Virus members from the families comprising the *Picornavirales* plus *Caliciviridae* are included to illustrate the broad inter-relationship of Electric ant solinvivirus, Electric ant virus 1, and Solenopsis invicta virus 10 in electric ant among these families. Legend for the number of substitutions per site is shown at the bottom left and GenBank accession numbers follow each virus name. ORFs are illustrated as blocks relative to the genome (line), and their position relative to the genome represents the reading frame (below line = reading frame [rf] 1; mid-line = rf 2; above line = rf 3)

The EASV (proposed species *Invictavirus electrico*) genome was 11,346 nts excluding the polyadenylated 3' terminus with a single large ORF in the sense direction (Fig. 4). The translated sequence of the predicted ORF exhibited identity with helicase (aa 609–716), cysteine protease (aa 1450–1602), RdRp (aa 2077–2366), and capsid (aa 2500) proteins of virus members in the *Solinviviridae*. Phylogenetic analysis of the RdRp placed EASV within the new *Solinviviridae* family (Fig. 4). EASV was most closely related (99% bootstrap support) to Solenopsis invicta virus 3, a virus of the *Invictavirus* genus.

### Electric ant virus 1 and Solenopsis invicta virus 10 in electric ant (EAV1 and SINV10 in EA)

The EAV1 genome was 10,233 nucleotides in length, excluding the polyadenylated 3' terminus. A single ORF in the sense direction was predicted with untranslated regions at the 5' and 3' ends (Fig. 4). Helicase (aa 323–425), cysteine protease (aa 1333), and RdRp (aa 1496–1880) sequence identities were detected in the 5'-proximal region of the ORF. No significant sequence similarity was detected from the 3' region. The polyprotein sequence exhibited the most significant Blast alignment identities to viruses from Hymenopteran insect hosts, including Solenopsis invicta virus 7 (ant; 99% genome coverage: 59% sequence identity), Milolii virus (ant; 98% genome coverage: 35% sequence identity), Apis picorna-like virus 5 (bee; 99% genome coverage: 45% sequence identity), and Bundaberg bee virus 8 (bee; 85% genome coverage: 35% sequence identity). Phylogenetic analysis of the RdRp placed EAV1 within a unique clade, separate from known *Picornavirales* families (Fig. 4). While group support for EAV1 was strong, larger taxonomic placement within the *Picornavirales* was uncertain.

The SINV10 in EA genome was 10,979 nucleotides in length, excluding the polyadenylated 3' terminus. A single ORF in the sense direction was predicted with untranslated regions at the 5' and 3' ends (Fig. 4). Helicase (aa 575–655) and RdRp (aa 1931–2327) sequence identities were detected in the 5'-proximal region of the ORF. Blastx analysis of the ORF revealed that this sequence exhibited 100% genome coverage and shared 99% amino acid sequence identity with Solenopsis invicta virus 10, which was previously identified from the red imported fire ant, *Solenopsis invicta* [42]. Significant sequence identity was also observed with Hubei picorna-like virus 54 from a Myriapod metagenome (99% genome coverage: 53 sequence identity) [36] and Riptortus pedestris virus 1 from the bean bug, *Riptortus pedestris* (83% genome coverage: 30% sequence identity) [42]. These viruses are not classified currently and may form a new virus taxon. Phylogenetic analysis placed SINV10 in EA nearest the *Solinviviridae* with moderate bootstrap support (59%). However, a jelly roll domain was not detected, which is observed in other *Solinviviridae* genomes. Thus, this group of virus sequences (Fig. 4) exhibits significant divergence from members of the picorna-like virus superfamily and may represent a unique taxonomic group.

### Electric ant rhabdovirus (EARV)

**Fig. 5:**
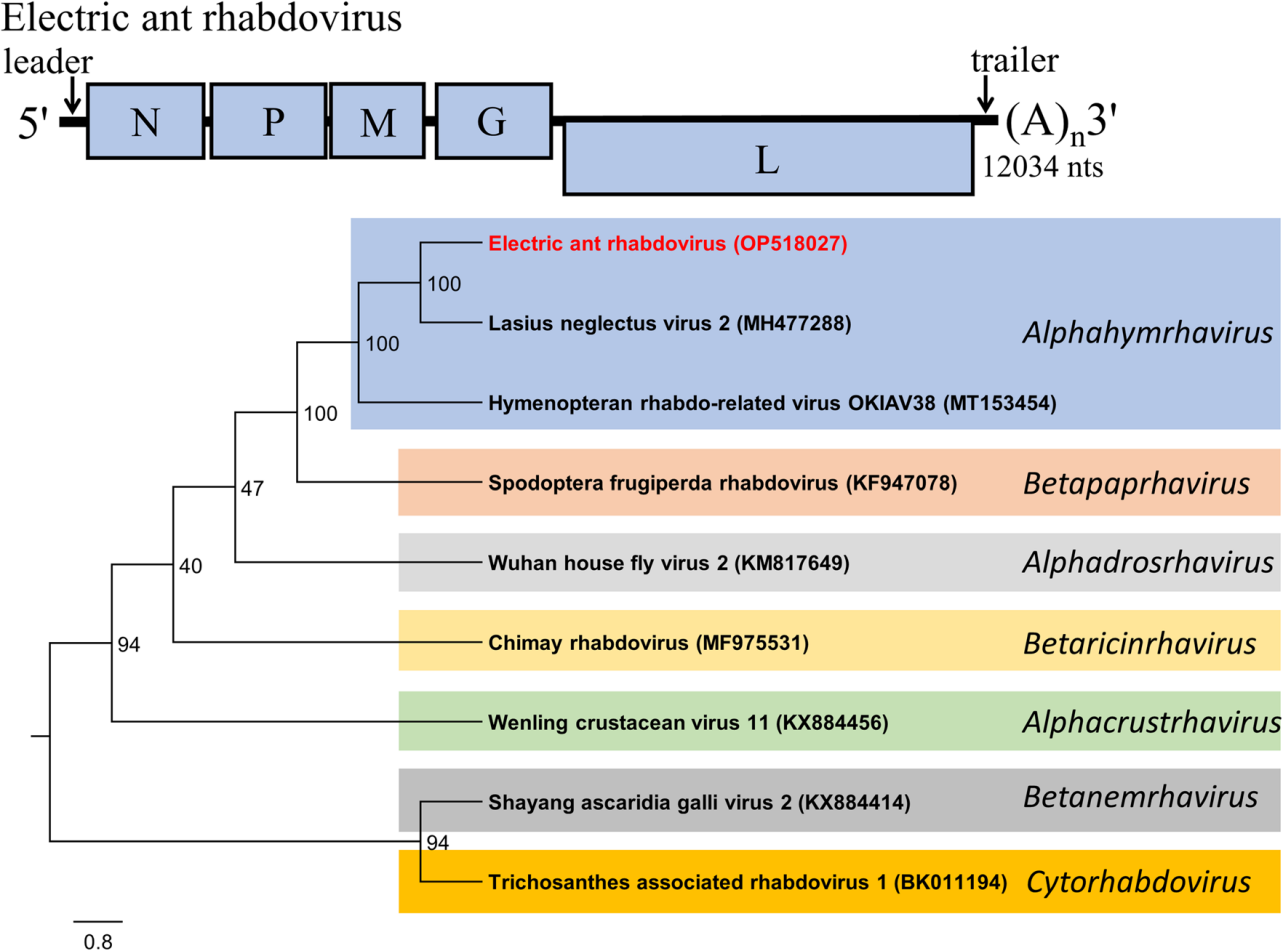
Diagrammatic representation of the predicted antigenome map of Electric ant rhabdovirus (top). Phylogenetic tree (unrooted) was generated using the amino acid sequence from the predicted “L” ORF (replication proteins). Numbers on the internal nodes represent the bootstrap values (500 replicates). Virus members from genera of related rhabdoviruses and top Blastp returns are included in the analysis. Legend for the number of substitutions per site is shown at the bottom left and GenBank accession numbers follow each virus name. ORFs are illustrated as blocks relative to the genome (line), and their position relative to the genome represents the reading frame (below line = reading frame [rf] 1; mid-line = rf 2; above line = rf 3)

The EARV (proposed species *Alphahymrhavirus electrico*) sequence was 12,034 nts (Fig. 5). The 5' leader antigenome sequence was 91 nts and 3' trailer sequence was 165 nts. EARV follows the typical genome structure and components of the *Rhabdoviridae*, ORFN (nucleocapsid; 470 aa), ORFP (polymerase cofactor; 450 aa), ORFM (RNA transcription regulation; 249 aa), ORFG (surface glycoprotein involved in endocytosis; 496 aa), and ORFL (RdRp and other replication/transcription functions; 2111 aa) (Fig. 5). Phylogenetic analysis of ORFL grouped EARV within the *Rhabdoviridae* family, and EARV appears closely related to Lasius neglectus virus 2 (97% genome coverage: 43% sequence identity) within the *Alphahymrhavirus* genus of negative sense viruses infecting hymenopteran insects [18].

### Replicative strand detection

**Fig. 6:**
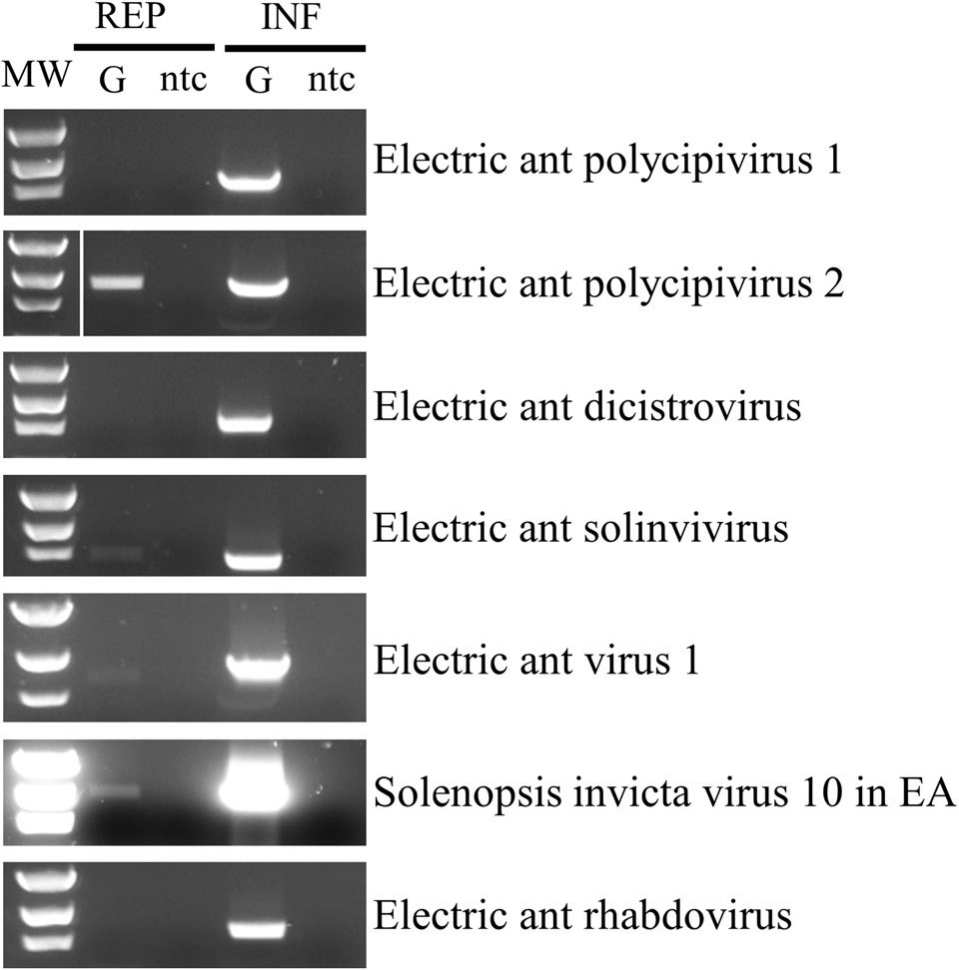
Tagged RT-PCR amplification of RNA from *Wasmannia auropunctata* to detect the replicative (REP) and infective (INF) genome strands of Electric ant polycipivirus 1 and 2, Electric ant dicistrovirus, Electric ant solinvivirus, Electric ant virus 1, Solenopsis invicta virus 10 in electric ant (EA), and Electric ant rhabdovirus. (G = genome, ntc = non-template control)

The replicative genome strands of EAPV2, EASV, EAV1, and SINV10 in EA were detected in *W. auropunctata* indicating that the ant was a suitable host for these viruses (Fig. 6). Conversely, the replicative genome strand of EAPV1, EADV, or EARV was not detected in *W. auropunctata*.

### Prevalence in wild populations

**Fig. 7:**
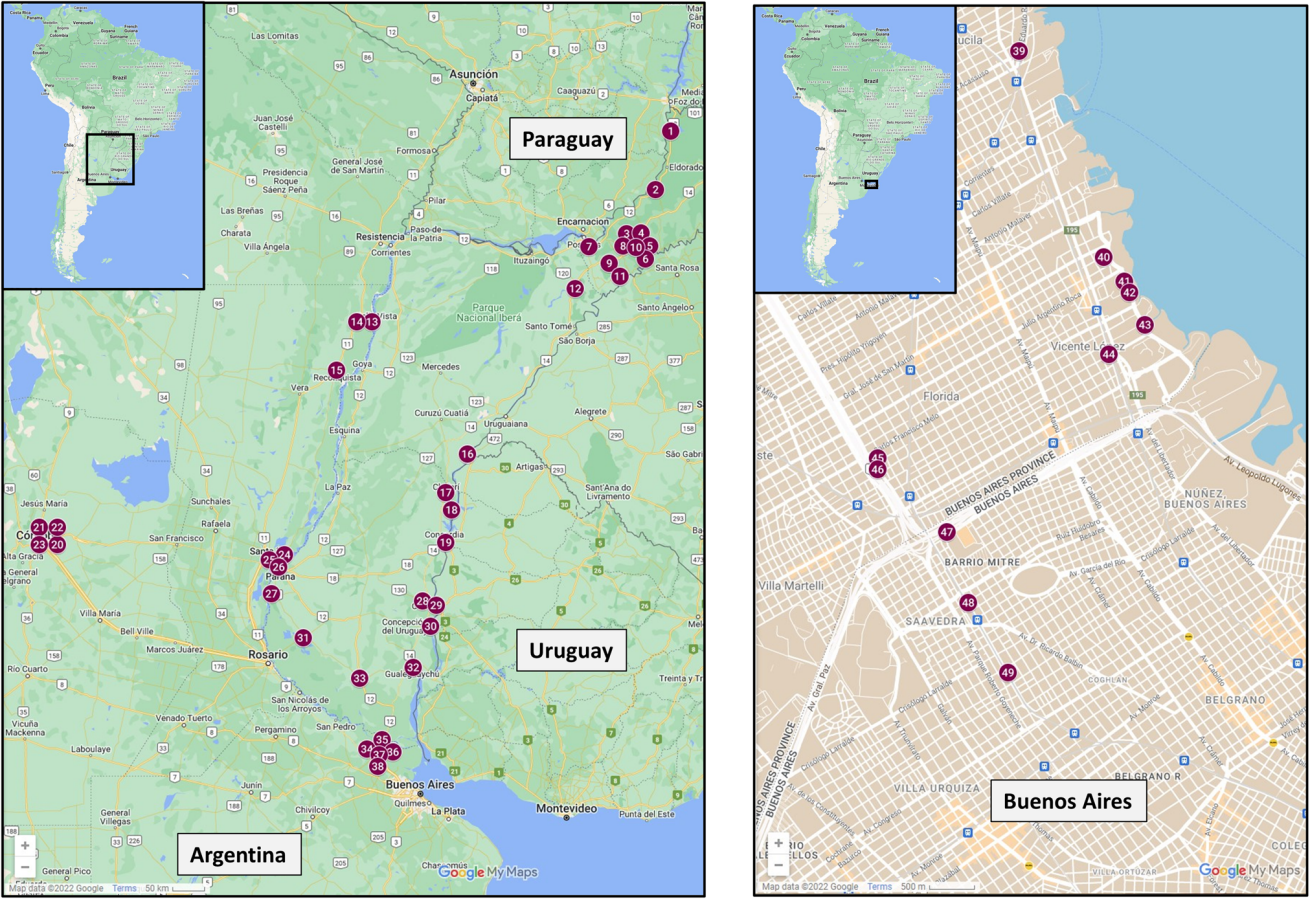
Map summarizing field collections of *Wasmannia auropunctata* made across Argentina. Buenos Aires is expanded and shown in the right pane. Each site number corresponds to a collection site and matches the sites listed in Table 2. The virus sequences detected and collection data for each site are also provided in Table 2

Field surveys were completely consistent with results from the library sequencing, and all virus genome sequences were detected in *W. auropunctata* collected only from Argentina (Table 2, Fig. 7). None of the virus genomes were detected in limited pooled samples of *W. auropunctata* collected from Queensland, Australia (*n* = 10), Pahoa, Hawaii, USA (*n* = 2), Hilo, Hawaii, USA (*n* = 4), Papaikou, Hawaii, USA (*n* = 2), Makiki, Hawaii, USA (*n* = 16), Gainesville, Florida, USA (*n* = 10), and Fort Lauderdale, Florida, USA (*n* = 3). Supplementary Table 4 contains the collection and other related metadata for the USA samples. Among the Argentinean field samples of *W. auropunctata* EARV was the most prevalent virus sequence detected (71.4%), followed by EASV (32.7%), EAPV1 (12.2%), EAPV2 and EAV1 (10.2%), and EADV (4.1%). Multiple virus infections were observed in numerous Argentinean *W. auropunctata* samples where up to 3 virus sequences were observed in individual ant samples.

**Table 2:**
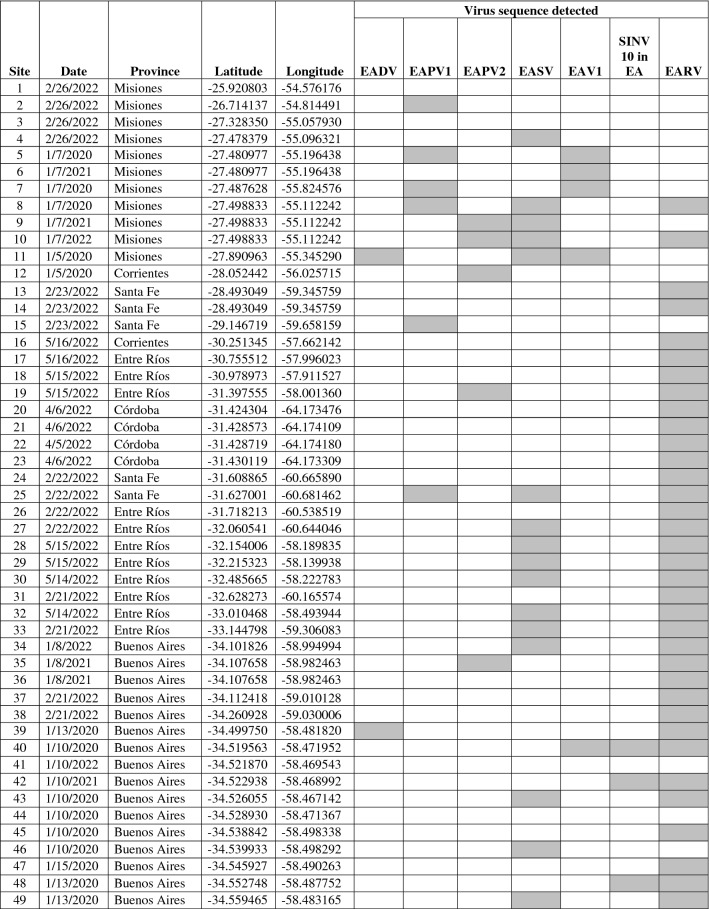
Geographic summary of virus sequences detected in field collected *Wasmannia auropunctata* worker ants collected from across Argentina

Each sample was comprised of a pooled group of 10–20 worker ants. Positive responses for each virus sequence are indicated (gray shading). Site numbers refer to locations illustrated in Fig. 7. Key to viruses: EADV = Electric ant dicistrovirus; EAPV1 = Electric ant polycipivirus 1; EAPV2 = Electric ant polycipivirus 2; EASV = Electric ant solinvivirus; EAV1 = Electric ant virus 1; SINV10 in EA = Solenopsis invicta virus 10 in electric ant; EARV = Electric ant rhabdovirus

Distribution in Argentina did not appear to be defined geographically for most of the virus sequences (Table 2, Fig. 7). EADV, EAPV1, EAPV2, EASV, and EAV1 were detected widely from the city of Buenos Aires in the south to El Dorado, Misiones, in the north. SINV10 in EA was only detected in samples collected in Buenos Aires and EARV, the most common virus sequence was only detected in ants south of latitude -28.493049.

## Discussion 

The objective of this research was to examine the virome and identify potential viral natural control agents of *W. auropunctata*. Six positive sense, single-stranded RNA virus genomes and one negative sense, single-stranded RNA virus genome have been identified and sequenced in entirety from transcriptome libraries of native Argentinean-derived *W. auropunctata*. The positive sense, single-stranded RNA virus genomes included one dicistrovirus (virus name: Electric ant dicistrovirus; species name: *Triatovirus electrico*), two polycipiviruses (Electric ant polycipivirus 1; *Sopolycivirus calcatterai* and Electric ant polycipivirus 2; *Sopolycivirus riversi*), one solinvivirus (Electric ant solinvivirus; *Invictavirus electrico*), and two genome sequences that were phylogenetically distinct and assorted with unclassified virus taxa (Electric ant virus 1; *Electric ant virus 1*, Solenopsis invicta virus 10 in electric ant; which is most likely Solenopsis invicta virus 10). The negative sense, single-stranded RNA virus genome was a rhabdovirus (Electric ant rhabdovirus; *Alphahymrhavirus electrico*).

EAPV1 and EAPV2 expand membership of the *Sopolycivirus* genus within the *Polycipviridae*. This is a new family of viruses within the *Picornavirales* with a unique polycistronic genome architecture [33]. Consistent with other members of this genus, EAPV1 and EAPV2 were detected in an ant host. The *Sopolycivirus* genus is comprised almost entirely of virus species that infect ants [33]. Representatives of this group have been shown to negatively impact their hosts. For example, within the fire ant *Solenopsis invicta*, the polycipivirus Solenopsis invicta virus 2 has been reported to cause significant reductions in fecundity, longer claustral periods, and slower growth of newly established *S. invicta* colonies [27].

Two of the virus genomes, EAV1 and SINV10 in EA were divergent and did not cluster with any established virus taxa. Blastp analysis of the RdRp of EAV1 identified close relatives including the Milolii virus from ghost ants (MF155030), the Alber virus (from an unknown species of ant from Lebanon [Alex Greninger—personal communication]; KX580900), Solenopsis invicta virus 7 from the red imported fire ant (MH719200), and the Bundaberg bee virus 8 from the honeybee (MG995704). Interestingly, this potentially new family is composed entirely of viruses from Hymenopteran hosts.

Solenopsis invicta virus 10 in electric ant is most certainly SINV10 identified previously from the fire ant, *S. invicta* [42]. The replicative genome of SINV10 in EA was detected in *W. auropunctata*, which indicates that this ant serves as host. Tests to examine the ability of SINV10 to replicate in *S. invicta* were not conducted [42] so it is not known if *S. invicta* is a true host to SINV10. Thus, the true host (or host range) of SINV10 cannot be ascertained currently.

*W. auropunctata* is omnivorous [44] so we were keenly aware that a virus sequence derived from a transcriptome may have originated from another organism that had been ingested. However, all seven of the virus genome sequences were highly expressed across the entire genome (Supplementary Fig. 2) suggesting that replication was occurring as would be expected with active viral infections (as opposed to ingestion of a relatively small number of packaged virus particles). In addition, the replicative genome of four of the viruses (EAPV2, EASV, EAV1, and SINV10 in EA) was detected in *W. auropunctata*, which indicates that the ant most assuredly serves as their host [5]. Failure to detect the replicative strand could have been from evaluating an incorrect stage or developmental period. Thus, the electric ant may serve as host for EAPV1, EADV, or EARV, but the replicative strand was simply missed.

In Argentina, nearly all of the virus sequences were widely distributed across the country (Table 2, Fig. 7), which is composed of a mix of sexually and clonally reproducing populations [4]. It will be interesting to investigate whether both reproductive forms (clonal and sexual) can serve as host to these viruses. The maternally inherited symbiont *Wolbachia* was found to be limited largely to sexually reproducing *W. auropunctata* populations in its native range [4]; *Wolbachia* was rarely detected in clonal invasive and clonal native populations [35]. Loss of symbionts (like *Wolbachia*), parasites, and natural enemies has been observed in other introduced populations of various species of ants that may have facilitated their success in introduced areas [34, 37, 47]. Interestingly, *Wolbachia* infections that do not induce reproductive parasitism have been shown to offer protection against viral infections [14]. For example, *Drosophila melanogaster* flies infected with *Wolbachia* are less susceptible to mortality from RNA viruses [14]. Have clonal populations of *W. auropunctata* lost their *Wolbachia* symbionts because they no longer benefit from their presence regarding RNA virus infection? Future investigations into the relationships between *Wolbachia*, RNA viruses, and the reproductive form of *W. auropunctata* are anticipated.

While insecticides can be temporarily effective at reducing the population and impact of *W. auropunctata* [3, 13, 38], sustained control of this pest ant will certainly rely on natural enemies [45]. Being one of the worst invasive species in the world [26], it is surprising that only two natural control agents are known from *W. auropunctata* [23, 28]. Despite the unknown impact of the viruses described here, they offer an attempt to identify new natural control agents and a starting point to investigate them. Also demonstrated here and by others, the metagenomics method greatly accelerates the prospecting phase for discovery of virus natural enemies [25].

## Supplementary Information

Below is the link to the electronic supplementary material.

Supplementary Figure 1. Phylogenetic analysis of the RdRp region of new Wasmannia auropunctata virus genomes and those of the picorna-like superfamily identified by Koonin et al. [19]. Key to virus abbreviations, family, and accession numbers: AhV, Atkinsonella hypxylon virus, Partitivirdae, L39126; ALSV, Apple latent spherical virus, Secoviridae, NC030941.1; ANV, avian nephritis virus, Astroviridae, AB033998; APV, Acyrthosiphon pisum virus, Unassigned, NC003780.1; BaYMV, Barley yellow mosaic virus, Potyviridae, NC002990; BBWV 1, Broad bean wilt virus 1, Secoviridae, NC005289.1; BDRC, Bryopsis cinicola chloroplast replicon, Unclassified; BDRM, Bryopsis mitochondria-associated dsRNA; BWYV, Beet western yellows virus, Solemoviridae, NC004756; CHV1, Cryphonectria hypovirus 1, Hypoviridae, NC001492; CHV2, Cryphonectria hypovirus 2, Hypoviridae, NC003534; CHV3, Cryphonectria hypovirus 3, Hypoviridae, NC000960; CHV4, Cryphonectria hypovirus 4, NC006431; CPMV, Cowpea mosaic virus, Secoviridae/Comovirinae, NC003549.1; CPV, Cryptosporidium parvum virus-1, Partiviridae, GCA002868475; CRLV, Cherry rasp leaf virus, Secoviridae, NC006271.1;CrPV, Cricket paralysis virus, Dicistroviridae, NC003924.1; CtRLV, Carrot red leaf virus, Solemoviridae, NC006265; DCV, Drosophila C virus, Dicistroviridae, NC001834.1; DWV, Deformed wing virus, Iflaviridae, NC004830.2; EADS, Electric ant dicistrovirus, Dicistroviridae, OP518023; EAPV1, Electric ant polycipivirus 1, Polycipiviridae, OP518021; EAPV2, Electric ant polycipivirus 2, Polycipiviridae, OP518022; EASV, Electric ant solinvivirus, Solinviviridae, OP518024; EAV1, Electric ant virus 1, Unclassified, OP518025; EMCV, encephalomyocarditis virus, Picornaviridae, NC001479; FCCV, Fragaria chiloensis cryptic virus, Partitiviridae, NC009519; FCV, Feline calicivirus, Caliciviridae, GCA008767155; FGMV, Fusarium graminearum mycovirus, Unassigned, LC006128; FHV, felid herpesvirus 1, Nodaviridae, NC013590; FMDV, Foot-and-mouth disease virus, Picornaviridae, GCA008799075; GFLV, Grapevine fanleaf virus, Secoviridae/Comovirinae, NC003615.1; GGNNV, Greasy grouper nervous necrosis virus, Nodaviridae, AF318942; GLV, Giardia lamblia virus, Totiviridae, NC003555; HaRNAV, Heterosigma akashiwo RNA virus, Marnaviridae, NC005281.1; HAstV1, human astrovirus 1, Astroviridae, Z25771; HAV, Hepatitis A virus, Picornaviridae, KT229611; HcRNAV, Heterocapsa circularisquama RNA virus, Unclassified, NC007518; HRV1A, Heterocapsa circularisquama RNA virus, Picornaviridae, NC007518; IFV, infectious flacherie virus, NC003781.1; JP A, marine RNA virus JP-A, Unassigned, NC009757.1; JP B, Marine RNA virus JP-B, Unassigned, NC009758.1; KFV, Kelp fly virus, Unassigned, NC007619.1; LRV1, Leishmania RNA virus 1, Totiviridae, NC003601; LTSV, Lucerne transient streak virus, Solemoviridae, GCA000861405; MBV, Mushroom bacilliform virus, Barnaviridae, NC001633; NoV, Nodamura virus, GCA000847805; NrV, Neckar River virus, Unassigned, NC038927.1; NwV, Newbury virus, Caliciviridae, GCA000851625; OAstV1, ovine astrovirus 1, Astroviridae, Y15937, SmVB, Sclerophtora macrospora virus A, Unclassified, Go0081047; OPV, Ophiostoma partitivirus 1, Partitiviridae, NC038918; PLRV, Potato leafroll virus, GCA021461725; PnPV, Perina nuda virus, Iflaviridae, NC003113.1; PYFV, Parsnip yellow fleck virus, Secoviridae, NC003628.1; RasR1, Raphanus sativus cryptic virus 2, Partitiviridae, NC010343; RHDV, Rabbit hemorrhagic disease virus, Caliciviridae, NC001543; RiPV, Riptortus pedestris virus 1, Unassigned, NC031750.1; RsRNAV01, Rhizosolenia setigera RNA virus 01, Unassigned, NC018613.1; RTSV, Rice tungro spherical virus, Secoviridae, NC001632.1; SBMV, Southern bean mosaic virus, Solemoviridae, GCA000860745; SCPMV, Southern cowpea mosaic virus, Solemoviridae, NC001625; ScVL A, Saccharomyces cerevisiae virus L-A, Totiviridae, NC003745; SDV, satsuma dwarf virus-S58, Secoviridae, AB009958; SINV10 in EA, Solenopsis invicta virus 10 in electric ant, Unassigned, OP518026; SJNNV, striped jack nervous necrosis virus, Nodaviridae, NC003448; SmVA, Sclerophtora macrospora virus A, Unclassified, AB083060; SPMMV, sweet potato mild mottle virus, Potyviridae, NC003797; SssRNAV, Aurantiochytrium single-stranded RNA virus 01, Unassigned, NC007522.1; SV, Sapporo virus, Caliciviridae, GCA000849945; TAstV1, Astroviridae, Y15936; TEV, Tobacco etch virus, Potyviridae, NC001555; TRSV, Tobacco ringspot virus, Secoviridae/Comovirinae, NC005096.1; TrV, Triatomoa virus, Dicistroviridae, NC003783.1; TSV, Taura syndrome virus, Dicistroviridae, NC003005.1; TVV, Trichomonas vaginalis virus, Totiviridae, NC003824; WSMV, Wheat Streak Mosaic Virus, Potyviridae, NC001886. Supplementary file1 (TIF 1377 KB)

Supplementary Figure 2. Mapping of RNA-Seq reads from four groups of electric ant samples collected in Argentina (Arg1, Arg2, Arg3, and Arg4) to seven virus genomes. The X-axis presents virus genome length in kilobases (kb) and the Y-axis represents sequencing coverage. Arrows indicate libraries with genome-wide sequencing coverage.Supplementary file2 (TIF 2911 KB)

Supplementary file3 (DOCX 23 KB)

Supplementary file4 (DOCX 42 KB)

Supplementary file5 (DOCX 15 KB)

Supplementary file6 (DOCX 23 KB)
