# Supplementary figures and images for: RNA virus discoveries in the electric ant, Wasmannia auropunctata
Source: Virus Genes. 2023 Feb 2;59(2):276–89. doi: 10.1007/s11262-023-01969-1 (PMC10025213; doi:10.1007/s11262-023-01969-1)

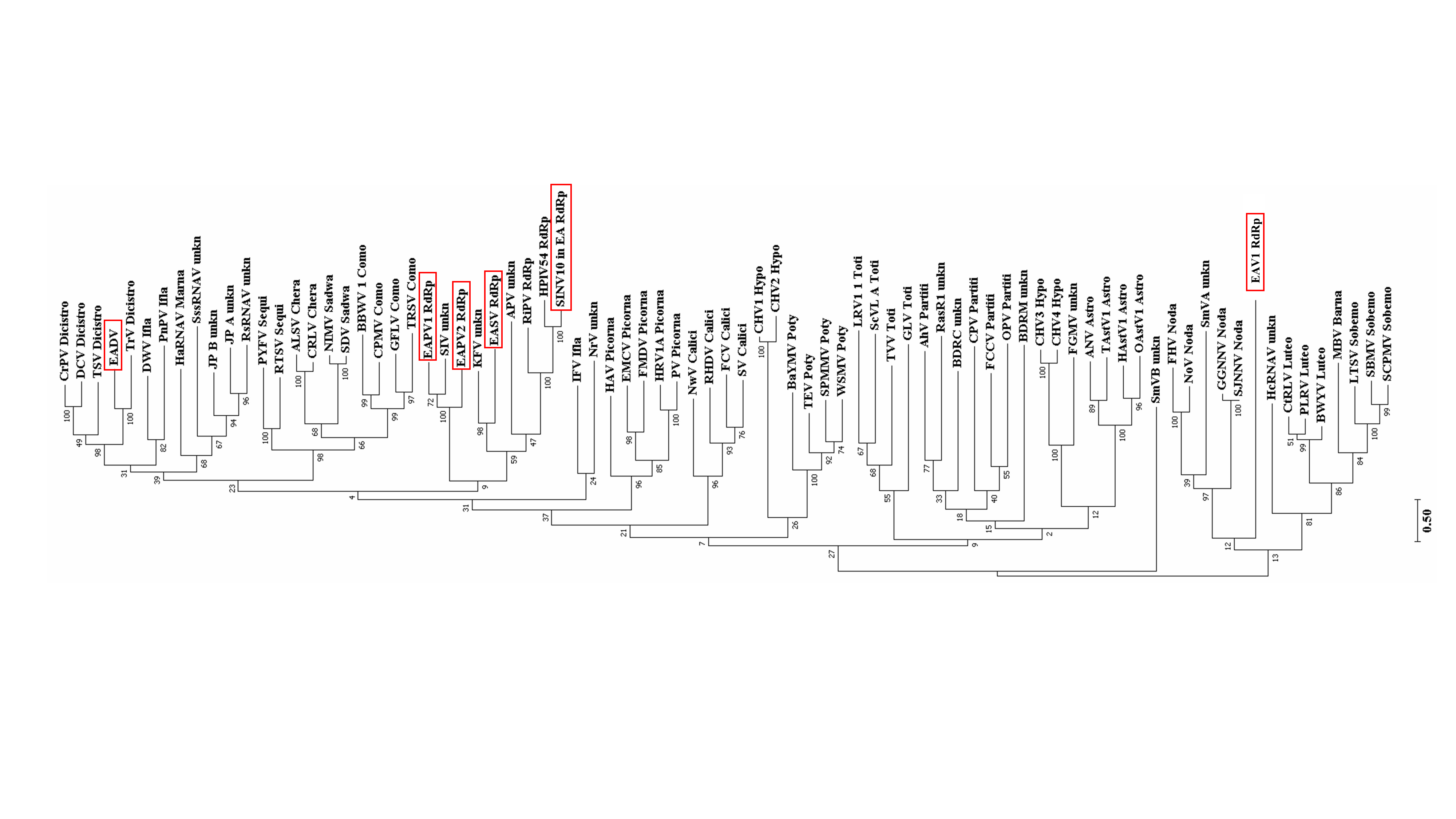

Supplement: Supplementary file 1 — Supplementary Figure 1. Phylogenetic analysis of the RdRp region of new Wasmannia auropunctata virus genomes and those of the picorna-like superfamily identified by Koonin et al. [19]. Key to virus abbreviations, family, and accession numbers: AhV, Atkinsonella hypxylon virus, Partitivirdae, L39126; ALSV, Apple latent spherical virus, Secoviridae, NC030941.1; ANV, avian nephritis virus, Astroviridae, AB033998; APV, Acyrthosiphon pisum virus, Unassigned, NC003780.1; BaYMV, Barley yellow mosaic virus, Potyviridae, NC002990; BBWV 1, Broad bean wilt virus 1, Secoviridae, NC005289.1; BDRC, Bryopsis cinicola chloroplast replicon, Unclassified; BDRM, Bryopsis mitochondria-associated dsRNA; BWYV, Beet western yellows virus, Solemoviridae, NC004756; CHV1, Cryphonectria hypovirus 1, Hypoviridae, NC001492; CHV2, Cryphonectria hypovirus 2, Hypoviridae, NC003534; CHV3, Cryphonectria hypovirus 3, Hypoviridae, NC000960; CHV4, Cryphonectria hypovirus 4, NC006431; CPMV, Cowpea mosaic virus, Secoviridae/Comovirinae, NC003549.1; CPV, Cryptosporidium parvum virus-1, Partiviridae, GCA002868475; CRLV, Cherry rasp leaf virus, Secoviridae, NC006271.1;CrPV, Cricket paralysis virus, Dicistroviridae, NC003924.1; CtRLV, Carrot red leaf virus, Solemoviridae, NC006265; DCV, Drosophila C virus, Dicistroviridae, NC001834.1; DWV, Deformed wing virus, Iflaviridae, NC004830.2; EADS, Electric ant dicistrovirus, Dicistroviridae, OP518023; EAPV1, Electric ant polycipivirus 1, Polycipiviridae, OP518021; EAPV2, Electric ant polycipivirus 2, Polycipiviridae, OP518022; EASV, Electric ant solinvivirus, Solinviviridae, OP518024; EAV1, Electric ant virus 1, Unclassified, OP518025; EMCV, encephalomyocarditis virus, Picornaviridae, NC001479; FCCV, Fragaria chiloensis cryptic virus, Partitiviridae, NC009519; FCV, Feline calicivirus, Caliciviridae, GCA008767155; FGMV, Fusarium graminearum mycovirus, Unassigned, LC006128; FHV, felid herpesvirus 1, Nodaviridae, NC013590; FMDV, Foot-and-mouth disease virus, Picornavi [file 11262_2023_1969_MOESM1_ESM.tif]

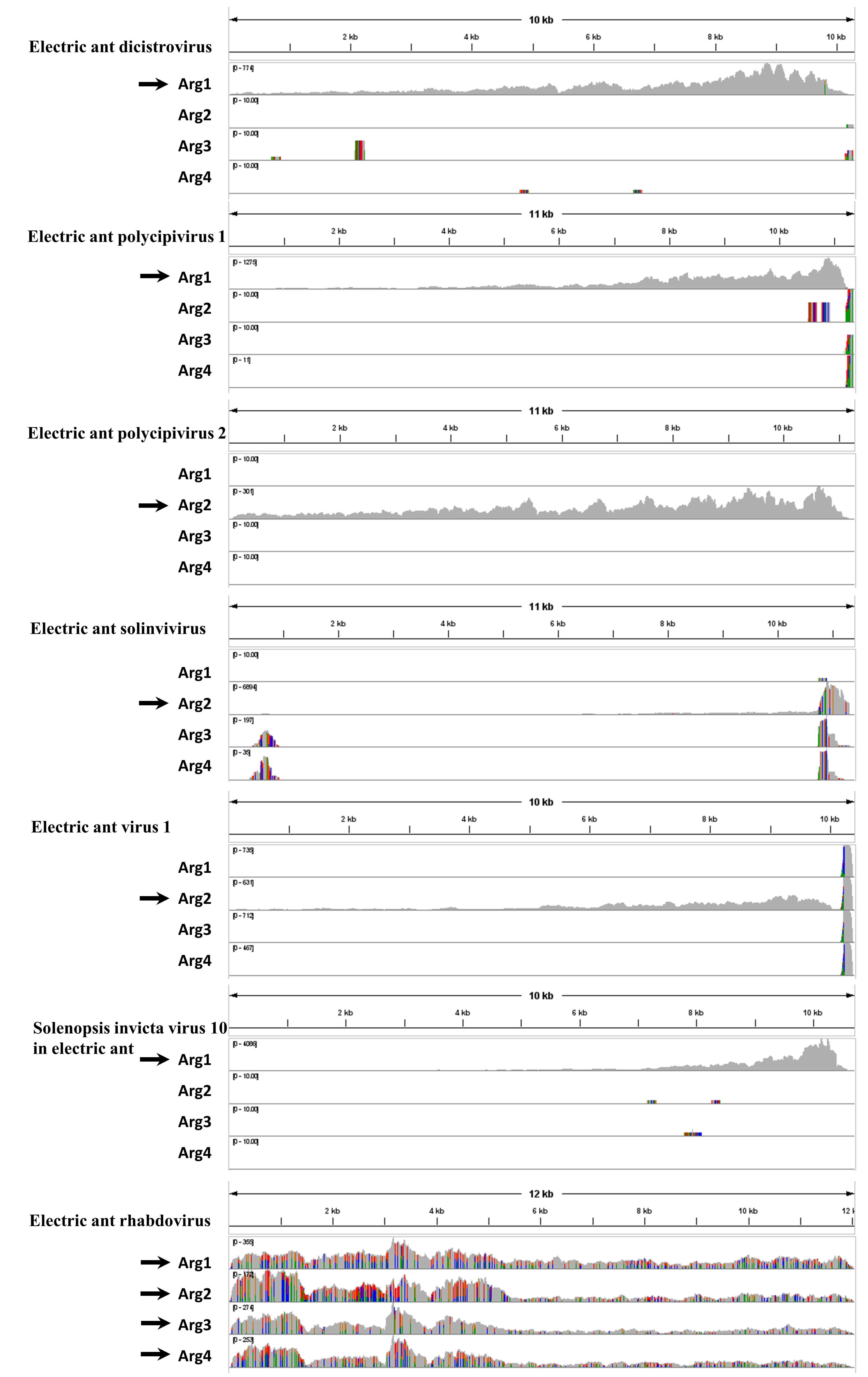

Supplement: Supplementary file 2 — Supplementary Figure 2. Mapping of RNA-Seq reads from four groups of electric ant samples collected in Argentina (Arg1, Arg2, Arg3, and Arg4) to seven virus genomes. The X-axis presents virus genome length in kilobases (kb) and the Y-axis represents sequencing coverage. Arrows indicate libraries with genome-wide sequencing coverage.Supplementary file2 (TIF 2911 KB) [file 11262_2023_1969_MOESM2_ESM.tif]
