# Supplementary material for: RNA virus discoveries in the electric ant, Wasmannia auropunctata
Source: Virus Genes. 2023 Feb 2;59(2):276–89. doi: 10.1007/s11262-023-01969-1 (PMC10025213; doi:10.1007/s11262-023-01969-1)
Supplement: Supplementary file 3 — Supplementary file3 (DOCX 23 KB) [file 11262_2023_1969_MOESM3_ESM.docx]

**Supplementary Table 1.** Metadata for *Wasmannia auropunctata* samples used in transcriptome library preparation.

| **Sample** | **Library** | **Collecting site** | **Province/City** | **Country** | **Date** | **Latitude** | **Longitude** | **Stage** |
| --- | --- | --- | --- | --- | --- | --- | --- | --- |
| **Was01** | **ARG1** | Train tracks, San Pedro | Buenos Aires | Argentina | 16-Sep-19 | -33.695507 | -59.680730 | Worker |
| **Was02** | **ARG1** | Route 1, Cayasta | Santa Fe | Argentina | 17-Sep-19 | -31.201095 | -60.157710 | Worker |
| **Was03** | **ARG1** | Route 1, Cayasta | Santa Fe | Argentina | 17-Sep-19 | -31.201095 | -60.157710 | Worker |
| **Was04** | **ARG1** | Route 1, Cayasta | Santa Fe | Argentina | 17-Sep-19 | -31.201095 | -60.157710 | Worker |
| **Was05** | **ARG1** | San Javier River, Romang | Santa Fe | Argentina | 18-Sep-19 | -29.499861 | -59.742652 | Worker |
| **Was06** | **ARG1** | Santa Ana, 20 km eastern Corrintes city | Corrientes | Argentina | 18-Sep-19 | -27.451850 | -58.651282 | Worker |
| **Was07** | **ARG1** | Near Candelaria | Misiones | Argentina | 19-Sep-19 | -27.472713 | -55.741645 | Worker |
| **Was08** | **ARG1** | Gobernador Virasoro | Corrientes | Argentina | 19-Sep-19 | -28.053361 | -56.015494 | Worker |
| **Was09** | **ARG1** | Villa Martelli, Vicente López | Buenos Aires | Argentina | 23-Sep-19 | -34.546184 | -58.495408 | Worker |
| **Was10** | **ARG1** | Saavedra neighborhood, Ciudad Autónoma de Buenos Aires | Ciudad Autónoma de Buenos Aires | Argentina | 23-Sep-19 | -34.559662 | -58.503328 | Worker |
| **Was11** | **ARG2** | Train station, Gobernador Virasoro | Corrientes | Argentina | 05-Jan-2020 | -28.052442 | -56.025715 | Worker |
| **Was12** | **ARG2** | Ruinas de Santa María La Mayor, Santa María | Misiones | Argentina | 05-Jan-2020 | -27.890963 | -55.345290 | Worker |
| **Was13** | **ARG2** | Arroyo Pindapoy, Garupá | Misiones | Argentina | 07-Jan-2020 | -27.487628 | -55.824576 | Worker |
| **Was14** | **ARG2** | Salto Berrondo, Oberá | Misiones | Argentina | 07-Jan-2020 | -27.480977 | -55.196438 | Worker |
| **Was15** | **ARG2** | Salto Berrondo, Oberá | Misiones | Argentina | 07-Jan-2021 | -27.480977 | -55.196438 | Worker |
| **Was16** | **ARG2** | Parque de Las Naciones, Oberá | Misiones | Argentina | 07-Jan-2020 | -27.498833 | -55.112242 | Worker |
| **Was17** | **ARG2** | Parque de Las Naciones, Oberá | Misiones | Argentina | 07-Jan-2021 | -27.498833 | -55.112242 | Worker |
| **Was18** | **ARG2** | Parque de Las Naciones, Oberá | Misiones | Argentina | 07-Jan-2021 | -27.498833 | -55.112242 | Worker |
| **Was19** | **ARG2** | Camping Las Tejas, Isla Talabera, Zárate | Buenos Aires | Argentina | 08-Jan-2020 | -34.107658 | -58.982463 | Worker |
| **Was20** | **ARG3** | Camping Las Tejas, Isla Talabera, Zárate | Buenos Aires | Argentina | 08-Jan-2021 | -34.107658 | -58.982463 | Worker |
| **Was21** | **ARG3** | Camping Las Tejas, Isla Talabera, Zárate | Buenos Aires | Argentina | 08-Jan-2021 | -34.101826 | -58.994994 | Worker |
| **Was22** | **ARG3** | Gral Las Heras 473, Paseo de la Costa, Vicente López | Buenos Aires | Argentina | 10-Jan-2020 | -34.526055 | -58.467142 | Worker |
| **Was23** | **ARG3** | Gral Las Heras 550, Paseo de la Costa, Vicente López | Buenos Aires | Argentina | 10-Jan-2021 | -34.522938 | -58.468992 | Worker |
| **Was24** | **ARG3** | Gral Las Heras 800, Paseo de la Costa, Vicente López | Buenos Aires | Argentina | 10-Jan-2021 | -34.521870 | -58.469543 | Worker |
| **Was25** | **ARG3** | Juan Díaz de Solis 1434, Paseo de la Costa, Vicente López | Buenos Aires | Argentina | 10-Jan-2020 | -34.519563 | -58.471952 | Worker |
| **Was26** | **ARG3** | Miguel de Azcuénaga 590, Vicente López | Buenos Aires | Argentina | 10-Jan-2020 | -34.528930 | -58.471367 | Worker |
| **Was27** | **ARG3** | Manuel Rosetti 854, Florida, Vicente López | Buenos Aires | Argentina | 10-Jan-2020 | -34.538842 | -58.498338 | Worker |
| **Was28** | **ARG3** | Blas Parera 723, Florida, Vicente López | Buenos Aires | Argentina | 10-Jan-2020 | -34.539933 | -58.498292 | Worker |
| **Was29** | **ARG4** | Eduardo Ramseyer 3246, La Lucila, Vicente López | Buenos Aires | Argentina | 13-Jan-2020 | -34.499750 | -58.481820 | Worker |
| **Was30** | **ARG4** | Eduardo Ramseyer 4088, La Lucila, Vicente López | Buenos Aires | Argentina | 13-Jan-2020 | -34.490350 | -58.481302 | Worker |
| **Was31** | **ARG4** | Eduardo Ramseyer 4036, La Lucila, Vicente López | Buenos Aires | Argentina | 13-Jan-2020 | -34.494885 | -58.481167 | Worker |
| **Was32** | **ARG4** | Plaza 3475 | Ciudad Autónoma de Buenos Aires | Argentina | 13-Jan-2020 | -34.559465 | -58.483165 | Worker |
| **Was33** | **ARG4** | Plaza 3531 | Ciudad Autónoma de Buenos Aires | Argentina | 13-Jan-2020 | -34.558698 | -58.483942 | Worker |
| **Was34** | **ARG4** | Plaza 3531 | Ciudad Autónoma de Buenos Aires | Argentina | 13-Jan-2020 | -34.558352 | -58.483672 | Worker |
| **Was35** | **ARG4** | Plaza 4171 | Ciudad Autónoma de Buenos Aires | Argentina | 13-Jan-2020 | -34.552748 | -58.487752 | Worker |
| **Was36** | **ARG4** | Warnes 916, Florida, Vicente López | Buenos Aires | Argentina | 14-Jan-2020 | -34.536222 | -58.494705 | Worker |
| **Was37** | **ARG4** | Santa Rosa y O´Higgins, Florida, Vicente López | Buenos Aires | Argentina | 14-Jan-2020 | -34.534188 | -58.494294 | Worker |
| **Was38** | **ARG4** | Vedia 3793, Vicente López | Buenos Aires | Argentina | 15-Jan-2020 | -34.545927 | -58.490263 | Worker |
| **Was39** | **ARG4** | Blas Parera 1400 y Urquiza, Florida, Vicente López | Buenos Aires | Argentina | 15-Jan-2020 | -34.534565 | -58.502490 | Worker |
| **Was40** | **FL1** | Field and fork gardens, University of Florida campus | Gainesville | United States | 12-Feb-20 | 29.644499 | -82.362876 | Worker |
| **Was41** | **FL1** | Field and fork gardens, University of Florida campus | Gainesville | United States | 12-Feb-20 | 29.644499 | -82.362876 | Worker |
| **Was42** | **FL1** | Field and fork gardens, University of Florida campus | Gainesville | United States | 12-Feb-20 | 29.644499 | -82.362876 | Worker |
| **Was43** | **FL1** | Field and fork gardens, University of Florida campus | Gainesville | United States | 12-Feb-20 | 29.644499 | -82.362876 | Worker |
| **Was44** | **FL1** | Field and fork gardens, University of Florida campus | Gainesville | United States | 12-Feb-20 | 29.644499 | -82.362876 | Worker |
| **Was45** | **FL1** | Field and fork gardens, University of Florida campus | Gainesville | United States | 12-Feb-20 | 29.644499 | -82.362876 | Worker |
| **Was46** | **FL2** | Field and fork gardens, University of Florida campus | Gainesville | United States | 12-Feb-20 | 29.644499 | -82.362876 | Queen |
| **Was47** | **FL2** | Field and fork gardens, University of Florida campus | Gainesville | United States | 12-Feb-20 | 29.644499 | -82.362876 | Queen |
| **Was48** | **FL2** | Field and fork gardens, University of Florida campus | Gainesville | United States | 12-Feb-20 | 29.644499 | -82.362876 | Queen |
| **Was49** | **FL2** | Field and fork gardens, University of Florida campus | Gainesville | United States | 12-Feb-20 | 29.644499 | -82.362876 | Queen |
| **Was50** | **HI1** | Papaikou Macnut Farm | Papaikou | United States | 30-Jan-20 | 19.787390 | -155.125111 | Worker |
| **Was51** | **HI1** | Papaikou Macnut Farm | Papaikou | United States | 30-Jan-20 | 19.787390 | -155.125111 | Worker |
| **Was52** | **HI1** | Papaikou Macnut Farm | Papaikou | United States | 30-Jan-20 | 19.787390 | -155.125111 | Worker |
| **Was53** | **HI1** | Papaikou Macnut Farm | Papaikou | United States | 30-Jan-20 | 19.787390 | -155.125111 | Worker |
| **Was54** | **HI1** | Papaikou Macnut Farm | Papaikou | United States | 30-Jan-20 | 19.787390 | -155.125111 | Worker |
| **Was55** | **HI2** | USDA Pacific Basin Agricultural Research Center grounds | Hilo | United States | 6-Feb-20 | 19.697361 | -155.094611 | Worker |
| **Was56** | **HI2** | USDA Pacific Basin Agricultural Research Center grounds | Hilo | United States | 6-Feb-20 | 19.697361 | -155.094611 | Worker |
| **Was57** | **HI2** | USDA Pacific Basin Agricultural Research Center grounds | Hilo | United States | 6-Feb-20 | 19.697361 | -155.094611 | Worker |
| **Was58** | **HI2** | USDA Pacific Basin Agricultural Research Center grounds | Hilo | United States | 6-Feb-20 | 19.697361 | -155.094611 | Worker |
| **Was59** | **HI2** | USDA Pacific Basin Agricultural Research Center grounds | Hilo | United States | 6-Feb-20 | 19.697361 | -155.094611 | Worker |
| **Was60** | **HI3** | Route 160, near the Captain Cook monument trail head | Captain Cook | United States | 10-Feb-20 | 19.456111 | -155.888667 | Worker |
| **Was61** | **HI3** | Route 160, near the Captain Cook monument trail head | Captain Cook | United States | 10-Feb-20 | 19.456111 | -155.888667 | Worker |
| **Was62** | **HI3** | Route 160, near the Captain Cook monument trail head | Captain Cook | United States | 10-Feb-20 | 19.456111 | -155.888667 | Worker |
| **Was63** | **HI3** | Route 160, near the Captain Cook monument trail head | Captain Cook | United States | 10-Feb-20 | 19.456111 | -155.888667 | Worker |
| **Was64** | **HI3** | Route 160, near the Captain Cook monument trail head | Captain Cook | United States | 10-Feb-20 | 19.456111 | -155.888667 | Worker |
