# Supplementary material for: RNA virus discoveries in the electric ant, Wasmannia auropunctata
Source: Virus Genes. 2023 Feb 2;59(2):276–89. doi: 10.1007/s11262-023-01969-1 (PMC10025213; doi:10.1007/s11262-023-01969-1)
Supplement: Supplementary file 4 — Supplementary file4 (DOCX 42 KB) [file 11262_2023_1969_MOESM4_ESM.docx]

**Supplementary Table 2.** Oligonucleotide primers used throughout this study. EASV (Accession OP518024) Electric ant solinvivirus; EAP1 (Accession OP518021) Electric ant polycipivirus 1; EAP2 (Accession OP518022) Electric ant polycipivirus 2; EADV (Accession OP518023) Electric ant dicistrovirus; EAV1 (Accession OP518025) Electric ant virus 1; SINV10 (Accession OP518026) Solenopsis invicta virus 10 in electric ant; EARV (Accession OP518027) Electric ant rhabdovirus.

| Virus | Primer Name | Sequence | Purpose |
| --- | --- | --- | --- |
| EASV | Wa1 | ATTCTTGATACCATAGTCGTATCCACTATAAAGA | Genome coverage |
| EASV | Wa2 | TTCAGGTTCGATTTTCATAGACTTATCTACATTAT | Genome coverage |
| EASV | Wa3 | ATTCGACAAACTCGAAGTGATTATGAGAAGA | Genome coverage |
| EASV | Wa4 | TCATTAGCGAGATCTCCAGTAAAGCCAGA | Genome coverage |
| EASV | Wa5 | CTTGGATTCTTGCTAAATTTGATGGAAGA | Genome coverage |
| EASV | Wa6 | TCATCACGAGTTACTTCAACAGTGGGA | Genome coverage |
| EASV | Wa7 | ATCATAATGATCCCTCAGGTCATCGTGAT | Genome coverage; Field evaluation |
| EASV | Wa8 | ATGGAATACTAAAGATTAATGCCGGAATAGG | Genome coverage; Field evaluation;  Replicating strand test |
| EASV | Wa9 | TTATTATACTGAGCCATATTTATATGAGGACCAAAT | Genome coverage |
| EASV | Wa10 | TTAGCCGCAATTTCAATTTGCTCATTTA | Genome coverage |
| EASV | Wa11 | CTGTATCTGGTAGAGGATTGGCTATGGATTATA | Genome coverage |
| EASV | Wa12 | ACGGAATAATCCATTAATTCATCTCGCTTA | Genome coverage |
| EASV | Wa13 | AACGTGTATATAAGGCTAGAGATATAAGTGTTTGGA | Genome coverage |
| EASV | Wa14 | TTGCTTATCAGAATTTGAAAGAACAATCATAGT | Genome coverage |
| EASV | Wa15 | ATGTATAGAGTAGTTGATATGGATTTCCCATCTAT | Genome coverage |
| EASV | Wa16 | TTCCATCTTCAGCATCTCATTAAAGGTTG | Genome coverage |
| EASV | Wa17 | TGTTATAAATTTGGATGCTACCGGCAATTA | Genome coverage |
| EASV | Wa18 | TTCTAACATATCGTGATATCCATGAAGCTTCTT | Genome coverage |
| EASV | Wa19 | AGTTGTCTGTTTCTGACAGGGTTGCG | Genome coverage |
| EASV | Wa20 | ATCTTCACGCGCAGTTGCTTTAGATGT | Genome coverage |
| EASV | Wa21 | AAGGATACTTGGATAGATGGACTTGCAA | Genome coverage |
| EASV | Wa22 | AGTCATCCAAAGTATAACTTCCAGTATCATCAG | Genome coverage |
| EASV | Wa23 | TCTTCCACAATTGGATATTCAAATAGCAATT | Genome coverage |
| EASV | Wa24 | TCAGCCTGAAGTATGAATCCATCTTGGA | Genome coverage |
| EASV | Wa25 | GATTTAAAGTTATGACAGATTCAGTATTGGAAGCA | Genome coverage |
| EASV | Wa26 | ATCTCTGCCTAAAACTAGATTGCTGTGC | Genome coverage |
| EASV | Wa27 | ACAGATTGATCATTGATAATGTTGTCAAACTTGATA | Genome coverage |
| EASV | Wa28 | AGCGATAGATTCATGATCTATCCTCTTCTGTC | Genome coverage |
| EASV | Wa29 | TGTGATTGATTCTTCAACTTATGGTGGCT | Genome coverage |
| EASV | Wa30 | AACAGCAGAGGAACTGCTGGAAGGTCC | Genome coverage |
| EASV | Wa31 | ATTCTTGATACCATAGTCGTATCCACTATAAAGA | 3’-RACE |
| EASV | Wa7TAG | GGCCGTCATGGTGGCGAATAAATCATAATGATCCCTCAGGTCATCGTGAT | Replicating strand test |
| EAPV2 | N78_1 | TAGCCCTAGCTACGTATGCCTGGTTCC | Genome coverage |
| EAPV2 | N78_2 | AGCGAAAGACACTCCTGGGCTATTGCTAGA | Genome coverage |
| EAPV2 | N78_3 | TGTGGTCATACCTGACTGATGTAGATTACTCA | Genome coverage |
| EAPV2 | N78_4 | TTCCTTACAAATGCCTCGTTTCAAGTGATC | Genome coverage |
| EAPV2 | N78_5 | TATTGCAATCAAACCACCAAGAGTTACTGG | Genome coverage |
| EAPV2 | N78_6 | ACTTTACCACGGAGCATAACAGCAATAAA | Genome coverage |
| EAPV2 | N78_7 | AGTTAGTCCCAATTCAGCTGCAGTGAATG | Genome coverage; Field evaluation |
| EAPV2 | N78_8 | ATATGAAGAACGCCATTGTGATTGTACTGTGC | Genome coverage; Field evaluation |
| EAPV2 | N78_9 | TCACCCGTATGAATGTCGATATGATGGAA | Genome coverage |
| EAPV2 | N78_10 | ATTCTGAGCATGATTGTTTGAATGATTGAAT | Genome coverage |
| EAPV2 | N78_11 | TTCAACTCGTGCATCTGGATTAGTAGAAACAT | Genome coverage |
| EAPV2 | N78_12 | ATAGCGAAATACGTCATCAATTATCATAGCCT | Genome coverage; Replicating strand test |
| EAPV2 | N78_13 | AATCCCGTTTGACCGAGATTGTAGTCAAGAA | Genome coverage |
| EAPV2 | N78_14 | TGTCTTCTTTCCACGACGATTCATAATTCGAG | Genome coverage |
| EAPV2 | N78_15 | TCATTGGGATGATCAGAATGATGGCATT | Genome coverage |
| EAPV2 | N78_16 | CCTGCGATGGAGATGCCAATGAA | Genome coverage |
| EAPV2 | N78_17 | TTATGCTCAAGCTCTTTGCACTGGTCAGTA | Genome coverage |
| EAPV2 | N78_18 | TTCATGCACGTAACAGTACGTGTATTTGGAGGA | Genome coverage |
| EAPV2 | N78_19 | CGAATCAAGGAAGAGTATGAGGAGTTTAGAT | Genome coverage |
| EAPV2 | N78_20 | AGTCAAAGATGTTCTTGACTCATGGAAATCA | Genome coverage |
| EAPV2 | N78_21 | AATGGATCTAAGTGTTGCCATGGATCTTCTA | 3’-RACE |
| EAPV2 | N78_22 | CCTCTCCATAATTTCTTCGGGTCGCTAA | 5’-RACE |
| EAPV2 | N78_24 | TGAAGATACGGAAAGCCGTTCAATTGC | 5’-RACE |
| EAPV2 | N78_11_TAG | GGCCGTCATGGTGGCGAATAATTCAACTCGTGCATCTGGATTAGTAGAAACAT | Replicating strand test |
| EAV1 | N124_1 | ATATAACCGAGAGTTATATGCACTGCCCACT | Genome coverage |
| EAV1 | N124_2 | TAGACACCATATGATTGACCACCATACGGT | Genome coverage |
| EAV1 | N124_3 | TGCGCCAGGAGTCGGTAAGTCAGAATT | Genome coverage |
| EAV1 | N124_4 | TGCTTCCTTAATTGCTGGTGGAATGCAA | Genome coverage |
| EAV1 | N124_5 | TATCTATGGTTGCATAGAGACACGTAATTCAGTAAT | Genome coverage |
| EAV1 | N124_6 | TGTTATCACCTTCACGATTCAATGCATCAGT | Genome coverage |
| EAV1 | N124_7 | AGCGTGCAAATGCTGCTCTTCCAA | Genome coverage; Field evaluation |
| EAV1 | N124_8 | ATGCCGAAGCATCTGTAACTCGGCT | Genome coverage; Field evaluation |
| EAV1 | N124_9 | TGGCTTTGAAGTCACGTCCTGTGTC | Genome coverage |
| EAV1 | N124_10 | ATACCAACGAAGCTGAGCTAATACTGATTCAA | Genome coverage |
| EAV1 | N124_11 | TCACAAGCTGATCTTATTGCTGATTCTGTACTT | Genome coverage |
| EAV1 | N124_12 | AGCAGTCCCAGGTTGAGCGAGGTA | Genome coverage; Replicating strand test |
| EAV1 | N124_13 | AGCTGAAGGAGAAACTGTCCGCTACAG | Genome coverage |
| EAV1 | N124_14 | ATGAGATATTGAATAGTCACTTCCGGCGTT | Genome coverage |
| EAV1 | N124_15 | ACCGAATGTCGTCCGCCCATAT | Genome coverage |
| EAV1 | N124_16 | TGTTACCATTACTGTAGATTCGCCCATCAT | Genome coverage |
| EAV1 | N124_17 | TCATGTTTCTTTAGCGGCGTTGGCA | Genome coverage |
| EAV1 | N124_18 | TCGTTCCGGGAGAAGGGTTGGGT | Genome coverage |
| EAV1 | N124_19 | TGGGCTCGGTTATGGAGAACCGC | Genome coverage |
| EAV1 | N124_20 | AATCCACCAGAAGGGTGGCGAATTATC | Genome coverage |
| EAV1 | N124_21 | ACCGATACTGGTTTATCCAGAGGACGTT | 3’-RACE |
| EAV1 | N124_22 | AGTGGGCAGTGCATATAACTCTCGGTTATAT | 5’-RACE |
| EAV1 | N124_24 | TTAGCGGTTACATGGGTTACGAGCTT | 5’-RACE |
| EAV1 | N124_11_TAG | GGCCGTCATGGTGGCGAATAATCACAAGCTGATCTTATTGCTGATTCTGTACTT | Replicating strand test |
| EAPV1 | N110_1 | GGGAAAGTATCAGGATCTTGATGGATCTAAT | Genome coverage |
| EAPV1 | N110_2 | TCACTGGCAGAAATACCACCTCCAAAGCTATTA | Genome coverage; 5’-RACE |
| EAPV1 | N110_3 | ATCCATCTTTGGACCATTCGGAGCCTT | Genome coverage |
| EAPV1 | N110_4 | TTTAGCAGGAACAACAAGAGGAGCCTGCT | Genome coverage |
| EAPV1 | N110_5 | AAGCATTTCTTCCAGTTTTCAAAGCCTGA | Genome coverage |
| EAPV1 | N110_7 | TAGGTGTTAGAGGAAGTATTGATGCACCTAA | Genome coverage; Field evaluation |
| EAPV1 | N110_8 | AGTCCGCGTACTATATGACCGCTCCGACT | Genome coverage; Field evaluation |
| EAPV1 | N110_9 | ACAAGAGCTATATGTGAGGTTGCCGAACCACT | Genome coverage |
| EAPV1 | N110_10 | TCTTTACATTCAACTCCATTAGCATAATCCAAGT | Genome coverage; Replicating strand test |
| EAPV1 | N110_11 | TGCTGATGGAGTGTATGTTGCACTTCTTCA | Genome coverage |
| EAPV1 | N110_12 | TTGCTCACTGAAGCAAGGACACCGCA | Genome coverage |
| EAPV1 | N110_13 | AGAACTTGGGAATTTTGAAGTTAGGATACAA | Genome coverage |
| EAPV1 | N110_14 | TCATCCATAACCACTTCAACTAGCATATGACGA | Genome coverage |
| EAPV1 | N110_15 | ATACTCCGGTGGTACTGCCAATGGCA | Genome coverage |
| EAPV1 | N110_16 | TCCGTTGATTGATACCAGACTGAGACGAGAAT | Genome coverage |
| EAPV1 | N110_17 | ACGTACTCAGGAACCACTTGCTATGCGA | Genome coverage |
| EAPV1 | N110_18 | TGCACAAATCAGAATGCTCCATTTCAACAA | Genome coverage |
| EAPV1 | N110_19 | ACACGTCATTCGTGACGTTACTCTGGAC | Genome coverage |
| EAPV1 | N110_20 | ATCCATCTTCTCGTGTTCCAGTAATGGT | Genome coverage |
| EAPV1 | N110_21 | AGAACCTTGTACTCGCTCTTTAGCAGTTCAA | Genome coverage |
| EAPV1 | N110_22 | TGGATTTGGAAGCCTCTATTTCTCCAGTT | Genome coverage |
| EAPV1 | N110_23 | TATGACAATATGGAAGCATTTCGGAAGCA | Genome coverage; 3’-RACE |
| EAPV1 | N110_24 | TCACGGAGAGGCCCAACTTCTATCAGCA | Genome coverage |
| EAPV1 | N110_30 | AACCACCAGGAACGCCTGAGTCAGATA | 5’-RACE |
| EAPV1 | N110_9_TAG | GGCCGTCATGGTGGCGAATAAACAAGAGCTATATGTGAGGTTGCCGAACCACT | Replicating strand test |
| EADV | N127Wa1 | TGGACGATCCCATGCGCAATTATT | Genome coverage |
| EADV | N127Wa2 | TTCACGAATTGTGATTCTCGCATATCGAAGTCA | Genome coverage |
| EADV | N127Wa3 | ACCATTGGAAAGGTTATAAAGGTAACCAGA | Genome coverage |
| EADV | N127Wa4 | TACCCTATACGACGCATAACACGATCGGTT | Genome coverage |
| EADV | N127Wa5 | TTGCCTGCAATGGTCGTGAATTCTATTATTTC | Genome coverage |
| EADV | N127Wa6 | TTGAGTAACTCGGACACATAAATCAAATCTCCT | Genome coverage |
| EADV | N127Wa7 | ATTTGGAACTGTTTGAAGTTATCCGTGCAGCTAAT | Genome coverage; Field evaluation |
| EADV | N127Wa8 | TGTATTGCCCATTTCAGATTCATGCTCACAA | Genome coverage; Field evaluation |
| EADV | N127Wa9 | AGTGGAGAAGTATGGACATCCATTGGCCAA | Genome coverage |
| EADV | N127Wa10 | CACCGCACTCAGCCGCTTGTGTAT | Genome coverage |
| EADV | N127Wa11 | AGGAATCCACTACTATAGATAATGAGGAAGGA | Genome coverage |
| EADV | N127Wa12 | ACAAACGAGTCTTGAACCACTTATGCAATGGTT | Genome coverage |
| EADV | N127Wa13 | TGAAGAAATCATTGAGGGATGTAGACAGG | Genome coverage |
| EADV | N127Wa14 | AGTTCACGTAAAGAATTGTCAATTTGTGCCTTAAC | Genome coverage |
| EADV | N127Wa15 | AGAACCGTTTAGGAAATTGGAAGATGTGTCCTATCT | Genome coverage |
| EADV | N127Wa16 | TATTCAATGTCATAGTTCGATTCCTAAACAATCCAGTA | Genome coverage; Replicating strand test |
| EADV | N127Wa17 | ATTTCGTTGGCTGGCTTCGGCGGCT | Genome coverage |
| EADV | N127Wa18 | TGGAGTGTCAGCATTAACTGTACAATCAATATCT | Genome coverage |
| EADV | N127Wa19 | TTAGTTGGTACTGCAGCAGATGAAACCGCAT | Genome coverage |
| EADV | N127Wa20 | GCCATTATTAGATGACCACCCATAGGCAT | Genome coverage |
| EADV | N127Wa21 | AATCCTGTTCAACAGATGGGTTTGCCGGAT | Genome coverage |
| EADV | N127Wa22 | GCATCCATCCCTGGATAGCTCTTGTTCGTGT | Genome coverage |
| EADV | N127Wa23 | TACCTTTGTGGGTATCGGAGTACAATGTA | Genome coverage |
| EADV | N127Wa24 | AATCCATATCATCTCCAGCTGCAACAGCA | Genome coverage |
| EADV | N127Wa25 | TGGTCCCATGGTATTGATAATCAATTTGATACA | Genome coverage |
| EADV | N127Wa26 | TTTTGCTGAAAGAAATCAAAAATCAAAGAAT | Genome coverage |
| EADV | N127Wa27 | ATGTGGGAGATTTAACAATAAACCAACGT | 3’-RACE |
| EADV | N127Wa30 | AATAATTGCGCATGGGATCGTCCA | 5’-RACE |
| EADV | N127Wa31 | TCTTATACACGTGTGAAGAAATAGATCACAACTA | 5’-RACE |
| EADV | N127Wa15TAG | GGCCGTCATGGTGGCGAATAAAGAACCGTTTAGGAAATTGGAAGATGTGTCCTATCT | Replicating strand test |
| SINV10 | SINV10_7 | CAAAGCGACCAGTGATCTTGGTTCTCGA | Field evaluation |
| SINV10 | SINV10_8 | AATCATTTGCTTGCGCTTCATGGTAGACATA | Field evaluation; Replicating strand test |
| SINV10 | N105_7_TAG | GGCCGTCATGGTGGCGAATAACAAAGCGACCAGTGATCTTGGTTCTCGA | Replicating strand test |
| EARV | N65_00 | ATTTATTATAGAAAGTACGCAGGACAAGAAAA | Genome coverage |
| EARV | N65_3 | TCCAGCAAATACTCCTGCCAAATATG | Genome coverage |
| EARV | N65_2 | AGAATTAATGCAGAGGCTGGATATCGCT | Genome coverage |
| EARV | N65_5 | AGTCCGGCAAGACTCTTAGAAGCATTGT | Genome coverage |
| EARV | N65_4 | AGAAGTAGGTGGACCTTCATCAGTCCTATCTATC | Genome coverage |
| EARV | N65_7 | AGGATAATGGGAAGATGGGAGTCAGACAATTC | Genome coverage |
| EARV | N65_6 | AGCTCCGGGTACAAACAACTCATTCT | Genome coverage; Field evaluation |
| EARV | N65_9 | CTAGGCCTGTTATACTCCCACTTATCGTTC | Genome coverage;  Field evaluation |
| EARV | N65_8 | CAAAAACTTAATCTTTGATGAATCTCCAGGC | Genome coverage |
| EARV | N65_11 | TCTGCACATACAGTAGATCATGATAACTGACA | Genome coverage |
| EARV | N65_10 | AACAAGAAGAGAAATACATAGATCAATATGTGGAC | Genome coverage |
| EARV | N65_13 | AGATTGAGAGATGTTGCTACTTTATGGATGTCTT | Genome coverage |
| EARV | N65_12 | ACTTAAGTGAGTTATATGGATTATATCGAATTTGG | Genome coverage |
| EARV | N65_15 | TTGTCTCAATCCTTCAATTCCTCCTAAATGA | Genome coverage |
| EARV | N65_14 | AAGCTGATGTTGCTCGGGCAAGGT | Genome coverage |
| EARV | N65_17 | AGCTCGGGATTCCACAGTTGCAG | Genome coverage |
| EARV | N65_16 | ACATAGGATCTGTTTTCCGCATCATATCTG | Genome coverage |
| EARV | N65_19 | TCTTTGTCTTGAGCTAGGTTCGGGTTCA | Genome coverage; Replicating strand test |
| EARV | N65_18 | TCAACCCCACTCTTGGGATCGACCA | Genome coverage |
| EARV | N65_21 | TGATAGACATTTATAGGATCCTGTAGTGGGA | Genome coverage |
| EARV | N65_20 | TCAGGTGGATTTACATTGGCAAATCTCCT | Genome coverage |
| EARV | F65_1 | TCTCTCAATTCAAATACTAGTGCAGCATGGAA | Genome coverage |
| EARV | F65_3 | CCTATCTAAAAGCTATGAGAAATATGAAAGGACA | Genome coverage |
| EARV | F65_5 | CAAGTCCATTGCCAACTGGAAGATTTGT | Genome coverage |
| EARV | F65_7 | CAATTTCAGTGCAGTTGGCTTCAGACTACCA | Genome coverage |
| EARV | F65_9 | AATACATCGCAGGAGACCCTTGGTCAATA | Genome coverage |
| EARV | F65_11 | GGACCTAGTACTGAAGCATGGAGGAA | Genome coverage |
| EARV | F65_13 | GGAGTGCTGCTTATCTATTGAATGGCAC | Genome coverage |
| EARV | F65_15 | CTTATGTTCAAACCTTTCTCAATCTATTAGAATCAGAA | Genome coverage |
| EARV | F65_17 | AAGTCATGCTGTGTACAAGAATTAGAGGACTCAAC | Genome coverage |
| EARV | F65_19 | TCCTAAATCTACTGAATTGCCTACAGGTGGA | Genome coverage |
| EARV | R65_2 | TCTGAGGAGTATTCTCTGGTGCATACCAGT | Genome coverage |
| EARV | R65_4 | TAAGGGAGACAATTCGTCCATCTTGA | Genome coverage |
| EARV | R65_6 | TGGATTATCATTAGAATCATCGTAGCAATATG | Genome coverage |
| EARV | R65_8 | TGTGATCCAGTTAGAGCTTATTCTAGAAGTGTAT | Genome coverage |
| EARV | R65_10 | TGAGAAGATACTGTTGAATGTGACATACATCA | Genome coverage |
| EARV | R65_12 | TTGTGTCTGTTATACGCACCGGAGTTACAAT | Genome coverage |
| EARV | R65_14 | CGCTGTCAAGTTAGCTGTGGCAGAGGA | Genome coverage |
| EARV | R65_16 | TGTACACTCAAACTCTTCCAAGCTTAATTCCAA | Genome coverage |
| EARV | R65_18 | TTCAGTACAGACTGGATTACCACTAACTCTAG | Genome coverage |
| EARV | R65_20 | GGATCTTCCCTGGCAAACTCTGCATCACAGAA | Genome coverage |
| EARV | N65_22 | TCTCTATACTTTGCTCCTATCCCATACAAAGC | 3’-RACE |
| EARV | R65_16_TAG | GGCCGTCATGGTGGCGAATAATGTACACTCAAACTCTTCCAAGCTTAATTCCAA | Replicating strand test |
|  | pTAG | GGCCGTCATGGTGGCGAATAA | Replicating strand test |
|  | Wa_Actin_F | GCCAGCACCTCCCTCGAGAAGAGCT | Field evaluation control |
|  | Wa_Actin_R | ACTCGTCGTATTCCTGTTTGGAGATCCACATCT | Field evaluation control |
