# Supplementary material for: RNA virus discoveries in the electric ant, Wasmannia auropunctata
Source: Virus Genes. 2023 Feb 2;59(2):276–89. doi: 10.1007/s11262-023-01969-1 (PMC10025213; doi:10.1007/s11262-023-01969-1)
Supplement: Supplementary file 5 — Supplementary file5 (DOCX 15 KB) [file 11262_2023_1969_MOESM5_ESM.docx]

**Supplementary Table 3.** Total output data sequencing summary.

| **Sample** | **Raw reads** | **Raw data (G)** | **Effective (%)** | **Error (%)** | **Q20 (%)** | **Q30 (%)** | **GC (%)** | **Mean insert (± SD)** | **Reads mapping to Formicidae (%)** |
| --- | --- | --- | --- | --- | --- | --- | --- | --- | --- |
| FL1 | 56180416 | 8.4 | 98.70 | 0.03 | 97.45 | 92.77 | 44.75 | 241.8 (43.1) | 67.4 |
| FL2 | 51628250 | 7.7 | 98.68 | 0.03 | 97.92 | 93.87 | 44.97 | 241.1 (43.8) | 74.2 |
| HI1 | 41454776 | 6.2 | 98.06 | 0.03 | 97.66 | 93.39 | 38.87 | 241 (41.1) | 52.5 |
| HI2 | 45042868 | 6.8 | 98.61 | 0.03 | 97.94 | 94.01 | 43.94 | 243.2 (39.9) | 67.3 |
| HI3 | 52035112 | 7.8 | 98.49 | 0.03 | 97.83 | 93.67 | 41.53 | 240.5 (43.2) | 62.8 |
| ARG1 | 50159734 | 7.5 | 98.44 | 0.03 | 97.72 | 93.34 | 42.17 | 238.8 (42.8) | 66.1 |
| ARG2 | 48122320 | 7.2 | 98.46 | 0.03 | 97.76 | 93.45 | 42.89 | 241.7 (41.7) | 67.5 |
| ARG3 | 44833922 | 6.7 | 98.17 | 0.03 | 97.96 | 93.96 | 43.29 | 240.2 (43.2) | 68.6 |
| ARG4 | 48797942 | 7.3 | 98.51 | 0.03 | 97.79 | 93.54 | 43.74 | 242.1 (41) | 69.3 |

Raw reads: Total amount of reads of raw data, each four lines taken as one unit. For paired-end sequencing, it equals the amount of read1 and read2.
Raw data: (Raw reads) * (sequence length), calculating in G. For paired-end sequencing like PE150, sequencing length equals 150, otherwise it equals 50 for sequencing like SE50.
Effective: (Clean reads/Raw reads) * 100%.
Error: Base error rate.
Q20, Q30: (Base count of Phred value > 20 or 30) / (Total base count).
GC: (G & C base count) / (Total base count).
