# Supplementary material for: RNA virus discoveries in the electric ant, Wasmannia auropunctata
Source: Virus Genes. 2023 Feb 2;59(2):276–89. doi: 10.1007/s11262-023-01969-1 (PMC10025213; doi:10.1007/s11262-023-01969-1)
Supplement: Supplementary file 6 — Supplementary file6 (DOCX 23 KB) [file 11262_2023_1969_MOESM6_ESM.docx]

**Supplementary Table 4.** Evaluation of Australian and USA field collected *Wasmannia auropunctata* worker ants for the presence of EAD = Electric ant dicistrovirus; EAP1 = Electric ant polycipivirus 1; EAP2 = Electric ant polycipivirus 2; EAS = Electric ant solinvivirus; EAV1 = Electric ant virus 1; SINV10 in EA = Solenopsis invicta virus 10 in electric ant; EAR = Electric ant rhabdovirus. All responses were negative. Metadata are included. Summary is shown in orange shading (ND = not detected).

| **Date** | **Country** | **Province/**  **City/State** | **Latitude** | **Longitude** | **Virus sequence** | | | | | | |
| --- | --- | --- | --- | --- | --- | --- | --- | --- | --- | --- | --- |
|  |  |  |  |  | **EADV** | **EAPV1** | **EAPV2** | **EASV** | **EAV1** | **SINV10 in EA** | **EARV** |
| 8/18/2021 | Australia | Stratford, Queensland | -16.872454 | 145.732349 |  |  |  |  |  |  |  |
| 8/18/2021 | Australia | Stratford, Queensland | -16.872605 | 145.731935 |  |  |  |  |  |  |  |
| 8/18/2021 | Australia | Stratford, Queensland | -16.872722 | 145.731784 |  |  |  |  |  |  |  |
| 8/18/2021 | Australia | Stratford, Queensland | -16.872802 | 145.731652 |  |  |  |  |  |  |  |
| 8/18/2021 | Australia | Stratford, Queensland | -16.873061 | 145.731200 |  |  |  |  |  |  |  |
| 8/18/2021 | Australia | Stratford, Queensland | -16.872075 | 145.732520 |  |  |  |  |  |  |  |
| 8/18/2021 | Australia | Stratford, Queensland | -16.872030 | 145.732549 |  |  |  |  |  |  |  |
| 8/18/2021 | Australia | Carovonica, Queensland | -16.864982 | 145.696694 |  |  |  |  |  |  |  |
| 8/18/2021 | Australia | Carovonica, Queensland | -16.864874 | 145.696770 |  |  |  |  |  |  |  |
| 8/18/2021 | Australia | Carovonica, Queensland | -16.865027 | 145.696675 |  |  |  |  |  |  |  |
| **Australia summary (percent infection for virus sequence)** ► | | | | | ND | ND | ND | ND | ND | ND | ND |
| 8/6/2021 | USA | Pahoa, Hawaii | 19.540944 | -154.850861 |  |  |  |  |  |  |  |
| 8/6/2021 | USA | Pahoa, Hawaii | 19.540944 | -154.850861 |  |  |  |  |  |  |  |
| 8/6/2021 | USA | Hilo, Hawaii | 19.681111 | -155.155556 |  |  |  |  |  |  |  |
| 8/6/2021 | USA | Hilo, Hawaii | 19.681111 | -155.155556 |  |  |  |  |  |  |  |
| 8/6/2021 | USA | Hilo, Hawaii | 19.681111 | -155.155556 |  |  |  |  |  |  |  |
| 8/6/2021 | USA | Hilo, Hawaii | 19.681111 | -155.155556 |  |  |  |  |  |  |  |
| 8/7/2021 | USA | Gainesville, Florida | 29.644499 | -82.362876 |  |  |  |  |  |  |  |
| 8/7/2021 | USA | Gainesville, Florida | 29.644595 | -82.362293 |  |  |  |  |  |  |  |
| 8/7/2021 | USA | Gainesville, Florida | 29.644628 | -82.361906 |  |  |  |  |  |  |  |
| 8/7/2021 | USA | Gainesville, Florida | 29.644555 | -82.361745 |  |  |  |  |  |  |  |
| 8/7/2021 | USA | Gainesville, Florida | 29.644645 | -82.361590 |  |  |  |  |  |  |  |
| 8/7/2021 | USA | Gainesville, Florida | 29.644718 | -82.362022 |  |  |  |  |  |  |  |
| 8/7/2021 | USA | Gainesville, Florida | 29.644727 | -82.362203 |  |  |  |  |  |  |  |
| 8/9/2021 | USA | Papaikou, Hawaii | 19.787389 | -155.125110 |  |  |  |  |  |  |  |
| 8/9/2021 | USA | Papaikou, Hawaii | 19.787389 | -155.125110 |  |  |  |  |  |  |  |
| 8/11/2021 | USA | Gainesville, Florida | 29.632405 | -82.307963 |  |  |  |  |  |  |  |
| 8/11/2021 | USA | Gainesville, Florida | 29.633056 | -82.307730 |  |  |  |  |  |  |  |
| 8/11/2021 | USA | Gainesville, Florida | 29.633044 | -82.307823 |  |  |  |  |  |  |  |
| 8/16/2021 | USA | Hilo, Hawaii | 19.697361 | -155.094611 |  |  |  |  |  |  |  |
| 8/16/2021 | USA | Hilo, Hawaii | 19.697361 | -155.094611 |  |  |  |  |  |  |  |
| 3/12/2022 | USA | Fort Lauderdale, Florida | 26.087690 | -80.177540 |  |  |  |  |  |  |  |
| 3/12/2022 | USA | Fort Lauderdale, Florida | 26.087690 | -80.177540 |  |  |  |  |  |  |  |
| 3/12/2022 | USA | Fort Lauderdale, Florida | 26.087690 | -80.177540 |  |  |  |  |  |  |  |
| 8/3/2021 | USA | Honolulu, HI | 21.30990 | -157.85800 |  |  |  |  |  |  |  |
| 8/3/2021 | USA | Honolulu, HI | 21.30990 | -157.85800 |  |  |  |  |  |  |  |
| 8/3/2021 | USA | Honolulu, HI | 21.30990 | -157.85800 |  |  |  |  |  |  |  |
| 8/3/2021 | USA | Honolulu, HI | 21.30990 | -157.85800 |  |  |  |  |  |  |  |
| 8/3/2021 | USA | Honolulu, HI | 21.30990 | -157.85800 |  |  |  |  |  |  |  |
| 8/3/2021 | USA | Honolulu, HI | 21.30990 | -157.85800 |  |  |  |  |  |  |  |
| 8/3/2021 | USA | Honolulu, HI | 21.30990 | -157.85800 |  |  |  |  |  |  |  |
| 8/3/2021 | USA | Honolulu, HI | 21.30990 | -157.85800 |  |  |  |  |  |  |  |
| 8/3/2021 | USA | Honolulu, HI | 21.30990 | -157.85800 |  |  |  |  |  |  |  |
| 8/3/2021 | USA | Honolulu, HI | 21.30990 | -157.85800 |  |  |  |  |  |  |  |
| 8/3/2021 | USA | Honolulu, HI | 21.30990 | -157.85800 |  |  |  |  |  |  |  |
| 8/3/2021 | USA | Honolulu, HI | 21.30990 | -157.85800 |  |  |  |  |  |  |  |
| 8/3/2021 | USA | Honolulu, HI | 21.30990 | -157.85800 |  |  |  |  |  |  |  |
| 8/3/2021 | USA | Honolulu, HI | 21.30990 | -157.85800 |  |  |  |  |  |  |  |
| 8/3/2021 | USA | Honolulu, HI | 21.30990 | -157.85800 |  |  |  |  |  |  |  |
| 8/3/2021 | USA | Honolulu, HI | 21.30990 | -157.85800 |  |  |  |  |  |  |  |
| **United States summary (percent infection for virus sequence)** ► | | | | | ND | ND | ND | ND | ND | ND | ND |
